# Activation of orphan receptor GPR132 induces cell differentiation in acute myeloid leukemia

**DOI:** 10.1038/s41419-022-05434-z

**Published:** 2022-11-27

**Authors:** Chunyang Yi, Jiacheng He, Dan Huang, Yumiao Zhao, Chan Zhang, Xiyun Ye, Ying Huang, Ruth Nussinov, Junke Zheng, Mingyao Liu, Weiqiang Lu

**Affiliations:** 1 Shanghai Key Laboratory of Regulatory Biology, Institute of Biomedical Sciences and School of Life Sciences, East China Normal University, Shanghai, 200241 China; 2 Department of Pathophysiology, Key Laboratory of Cell Differentiation and Apoptosis of Chinese Ministry of Education, Shanghai Jiao Tong University School of Medicine, Shanghai, China; 3 NMPA Key Laboratory of Rapid Drug Inspection Technology, Guangdong Institute for Drug Control, 766 Shenzhou Road, Guangzhou, 510663 China; 4 Computational Structural Biology Section, Basic Science Program, Frederick National Laboratory for Cancer Research, National Cancer Institute at Frederick, Frederick, MD 21702 USA; 5 Department of Human Molecular Genetics and Biochemistry, Sackler School of Medicine, Tel Aviv University, Tel Aviv, 69978 Israel

**Keywords:** Drug discovery, Cell biology

## Abstract

Blocked cellular differentiation is a critical pathologic hallmark of acute myeloid leukemia (AML). Here, we showed that genetic activation of the orphan GPCR GPR132 significantly induced cell differentiation of AML both in vitro and in vivo, indicating that GPR132 is a potential trigger of myeloid differentiation. To explore the therapeutic potential of GPR132 signaling, we screened and validated a natural product 8-gingerol (8GL) as a GPR132 agonist. Notably, GPR132 activation by 8GL promoted differentiation and reduced colony formation in human AML cell lines with diverse genetic profiles. Mechanistic studies revealed that 8GL treatment inhibits the activation of the mammalian target of rapamycin (mTOR), a regulator of AML cell differentiation blockade, via activating GPR132-G_s_-PKA pathway. We further showed that the combination of 8GL and an mTOR inhibitor synergistically elicited AML cell differentiation in vitro. Importantly, 8GL alone or in combination with an mTOR inhibitor remarkably impaired tumor growth and extended mouse survival in an AML xenograft model accompanied by enhanced cell differentiation. Notably, genetic or pharmacological activation of GPR132 triggered the differentiation of human primary AML cells. In summary, this study demonstrated that activation of orphan GPR132 represents a potential strategy for inducing myeloid differentiation in AML patients.

## Introduction

AML is the most common type of acute leukemia affecting adults, with an estimated number of new cases and deaths in the United States of 20,240 and 11,400, respectively, in 2021 [1, 2]. AML represents one of the most difficult-to-treat cancers, with a five-year relative survival rate of only 28.7% [3]. In the past three decades, the standard induction of chemotherapy, combining cytarabine and anthracycline, has been the first-line treatment for newly diagnosed AML cases. Although there is considerable remission following the original administration of these cytotoxic drugs, patient prognosis is still very poor, especially in elderly patients with a 5-year relative survival rate of only 3–10% [4–7]. Exploration of innovative targets may offer new therapeutic strategies to treat patients with refractory/resistant AML.

G protein-coupled receptors (GPCRs) are the largest and most diverse family of cell–surface proteins in humans [8]. These transmembrane receptors can transduce extracellular physiochemical stimuli into intracellular responses via their linked heterotrimeric G proteins [9]. GPCRs constitute the largest family of drug targets with approved therapeutics, and approximately 35% of marketed drugs achieve their therapeutic efficacy via GPCRs [10, 11]. Multiple GPCR-targeting drugs have been for the treatment of solid cancers, including prostate cancer, and pancreatic cancer [10, 12, 13]. However, the biological role of GPCRs in AML is not well defined.

Blocked cell differentiation is a critical pathologic feature of AML [14]. In this study, we showed that genetic activation of orphan GPR132 significantly induces differentiation and impairs cell growth in AML cell lines and human primary AML cells. We identified 8-gingerol (8GL) as a GPR132 agonist via screening a natural product library. We show that pharmacological activation of GPCR132 by 8-GL effectively triggers cell differentiation in AML by inactivating mTOR signaling. Our findings further demonstrated that a combination of 8-GL and mTOR inhibitor synergistically curbs tumor growth and extends mice survival in a systematic leukemia model.

## Methods

### Cell culture

HL60, MV4-11, Kasumi1, THP1, HEK293, and CHO cell lines were obtained from the Stem Cell Bank (Chinese Academy of Sciences, Shanghai, China). NB4 and OCI-AML3 cell lines were obtained from the American Type Culture Collection (ATCC). AML cell lines were maintained in RPMI 1640 medium supplemented with 10% heat-inactivated fetal bovine serum (FBS) and 1% penicillin/streptomycin solution (Invitrogen, Grand Island, NY). HEK293 cells were cultured in Dulbecco’s modified Eagle’s medium (DMEM), and CHO cells were cultured in DMEM/F12 medium. All cells were maintained in a humidified incubator with 5% CO_2_ at 37 °C. Cell lines were identified by STR profiling and were confirmed to be mycoplasma free.

### GPR132 Tet-On expression system

A tetracycline (tet)-induced gene expression system in AML cells was established using a Tet-On lentiviral vector (Clontech; Mountain View, CA, USA). Briefly, the full-length cDNA of *GPR132* in GPR132-Tango (Addgene, plasmid #66315) was subcloned into the pLVX-TetOne-Puro vector. HEK293 cells were transfected with Tet-On-GPR132, psPAX2 (Addgene, plasmid #12260), and pMD2.G (Addgene, plasmid #12259), and then the lentivirus supernatant was collected. Human AML cell lines were resuspended in a medium containing 70% lentivirus supernatant, 10% FBS, 20% fresh RPMI 1640 medium, and 1 μg/mL polybrene (Millipore, MA, USA). Subsequently, infected cells were subjected to puromycin selection (1 μg/mL). GPR132 expression was induced by treatment with 1 μM doxycycline.

### Knockout of GPR132 in AML cells

GPR132 knockout cell lines were established by CRISPR–Cas9 system [15]. Two distinct sgRNA sequences targeting fourth exon of GPR132 were designed using benchling (https://www.benchling.com/academic/): sg1 (5′-AGGGCCTCTAGGGAACCGTG-3′) and sg2 (5′-GGGGTGCCAGGAGTCCCCAG-3′). In brief, sgRNA sequence was subcloned into the lentiCRISPR v2 (addgene, #52961) and lentivirus supernatant was produced as described above. Totally, 5 × 10^6^ AML cells were suspended with 2 mL medium containing 70% lentivirus supernatant, 10% FBS, 20% fresh RPMI 1640 medium and 1 μg/mL polybrene, and then selected with 0.5 μg/mL puromycin.

### Cell viability and proliferation assay

Cell viability and proliferation assay were performed as described in our previous study [16]. Briefly, AML cells were seeded into 96-well plates (3 × 10^3^ per well) and treated with indicated concentrations of compounds for 72 h. Cell viability was then determined by MTS assay (Promega; Madison, WI), and optical density was measured at 490 nm using a Cytation^TM^ 5 Imaging Multi-Mode reader (BioTek, Winooski, VT). Cells were counted for proliferation determination. Cell viability and cell proliferation were uniforms as a percentage versus control (vehicle set at 100%).

### Drug combination analysis

The in vitro synergistic study was performed as described previously [16]. Briefly, AML cells were incubated with increasing concentrations of compounds alone or in combination for 72 h, and cell viability was measured by MTS assay. Synergy was evaluated by the bliss independence model via the SynergyFinder web application [17]. The combination index (CI) and fraction affected (Fa) of drug combination were calculated using CalcuSyn software Version 2.1 (Biosoft, Cambridge, UK) [18].

### Wright–Giemsa analysis

The cellular morphology of AML cell lines or human primary AML cells was assessed by Wright–Giemsa staining as described in our previous study [19].

### NBT reduction assay

Treated AML cell lines or human primary AML cells were incubated with 1 mg/mL nitro-blue tetrazolium (NBT) (Sigma) for 30 min at 37 °C and then smeared to coverslip followed by horizontal centrifugation of 10 s at 1500 rpm. The NBT formazan was imaged by microscope (Olympus) or quantified by measuring absorbance at 560 nm.

### Colony formation assay

We used the colony formation assay to assess the ability of cells to form colonies and to measure the proliferative potential of AML cells. A colony-forming unit (CFU) is defined as a viable clonogenic cell cluster with more than 50 cells. Colony formation assay was performed as described previously by using a methylcellulose-based medium Methocult^TM^ (H4434 or H4436, Stem Cell Technologies) [20]. In brief, human AML cell lines or human primary AML cells were seeded into a 24-well plate (1 × 10^3^ cells per well) in methylcellulose media and pre-treated with indicated concentrations of compounds at 37 °C. Cells were allowed to grow for 5–7 days, and formed colonies were photographed under an inverted microscope (BD Biosciences; San Jose, CA, USA). Formed CFU was counted and uninformed as a percentage versus control (vehicle set at 100%).

### Flow cytometry analysis

The cell surface markers of differentiation were evaluated by flow cytometry as described in our previous study [16]. AML cell lines or human primary AML cells were cultured in 6-well plates at a density of 1 × 10^6^ cells per well and treated with different compounds for 72 h. Subsequently, treated cells were resuspended in phosphate-buffered saline containing 1 mM EDTA and 2% FBS, and incubated with FITC-conjugated anti-CD11b or PE-conjugated anti-CD14 (BioLegend) for 30 min in the dark at room temperature. Flow cytometer data were collected by BD FACS Caliber (BD Biosciences, NJ, USA) and analyzed through FlowJo (BD Biosciences, NJ, USA).

### Western blotting

Treated cells were washed three times with PBS and lysed at 4 °C with radioimmunoprecipitation lysis buffer (RIPA) (150 mM NaCl, 1 mM EDTA, 100 mM Tris-HCl, 1% Triton X-100, 1% sodium deoxycholate, and 0.1% SDS) containing protease and phosphatase inhibitors (Roche; San Francisco, CA). Protein concentrations were measured using bicinchoninic acid assay (Thermo Fisher Scientific; Waltham, MA). Proteins were subjected to SDS-polyacrylamide gel electrophoresis and transferred to nitrocellulose membranes. Membranes were probed with specific primary antibodies overnight at 4 °C, followed by exposure to corresponding secondary antibodies. Full-length western blots were provided in the Supplemental Material.

### Quantitative real-time PCR (qRT-PCR)

RNA isolation and reverse transcription from tumor tissues were conducted by using TRIzol reagent kit (Invitrogen; Carlsbad, CA) and PrimeScript^TM^ RT reagent Kit (Takara, Beijing, China) according to the manufacturer’s protocol. Quantitative real-time PCR was performed using SYBR green reaction mixture in the ABI Q3 fast real-time PCR system (Applied Biosystems, USA). The gene expression was analyzed via the ΔΔCt method after normalization to GAPDH expression. The primers used in this paper were listed in Supplemental Table S4.

### Tango assay

Tango assay was performed as described in our previous study [21]. CHO cells cultured in a 6-cm dish were co-transfected with GPR132-Tango, ARRB2-TEV, and TRE-Luc with a ratio of 2:1:1 by using polyethyleneimine (PEI). Transfected cells were then seeded into a 96-well plate at a density of 2 × 10^4^ cells per well. After 24 h, the medium was refreshed, and cells were incubated with compounds at indicated concentrations for 18 h. Subsequently, cells were lysed with Passive Lysis buffer (Promega, Madison, WI, USA) for 15 min at room temperature in a shaker at 70 rpm. The cell lysate was transferred to white 96-well plates, and the bioluminescence was determined immediately after the addition of luciferase substrate (Promega, Madison, WI, USA) with a Cytation^TM^ 5 Imaging Multi-Mode reader (BioTek, Winooski, VT).

### CRE reporter assay

cAMP-responsive element (CRE) reporter assay was performed as described in our previous study with a minor modification [22]. CHO cells were co-transfected with GPR132 and CRE-Luc vector with a ratio of 1:1 and then seeded into 96-well plates (2 × 10^4^ cells per well). After overnight incubation, cells were treated with varied concentrations of compounds for 24 h. Luciferase activities were measured by Luciferase Assay System (Promega, Madison, WI, USA) using Cytation^TM^ 5 Imagine Multi-Mode reader.

### Nano-Bit^®^ β-arrestin recruitment assay

To detect ligand-induced interaction between GPR132 and β-arrestin, a NanoBit^®^ β-arrestin recruitment assay was performed [23]. HEK293T cells were co-transfected GPR132-LgBit and β-arrestin2-SmBit vectors at 1:1 and seeded into white 96-well plates (with transparent bottom) at a density of 2 × 10^4^ cells per well. Twenty-four hours later, cells were loaded with Nano-Glo Live Cell Reagent (Promega, Madison, WI, USA) in a CO_2_-independent medium at 37 °C. Then, compound stock solutions were dispensed to 96-well plates, and luminescence signals were monitored continuously for 1 h or more at 37 °C with a Cytation^TM^ 5 Imagine Multi-Mode Reader (BioTek, Winooski, VT).

### In vivo Models

Six-to-eight-week-old female BALB/c Nude mice and NSG mice were obtained from the National Rodent Laboratory Animal Resources (Shanghai, China). All animal treatments were conducted according to Institutional Animal Care and Use Committee guidelines and under an institutional protocol approved by East China Normal University. All mice were blinded to the group allocation during the experiments.

To examine the in vivo role of GPR132 in AML, 1 × 10^7^ Tet-on-GPR132 HL60 cells or control HL60 cells were subcutaneously injected into the mid-right flank of BALB/c Nude mice. When tumor volume reached 300 mm^3^, mice xenografted with Tet-on-GPR132 HL60 or control HL60 were randomized into 2 groups (6 mice/group), respectively, and administered with doxycycline (1 mg/mL Dox in drinking water) or vehicle for 2 weeks.

To evaluate the in vivo anti-AML activity of 8GL alone or in combination with mTOR inhibitor everolimus, 1 × 10^7^ HL60 cells were subcutaneously injected into the mid-right flank of BALB/c Nude mice. For 8GL alone, mice were randomized into 3 groups (7 mice per group) and intraperitoneally injected with 8GL (30 mg/kg and 60 mg/kg) or with the vehicle daily for continuous 2 weeks; for 8GL in combination with everolimus, mice were randomized into 4 groups (6 mice per group) and intraperitoneally injected with vehicle, 8GL (30 mg/kg) and/or everolimus (3 mg/kg) daily for two continuous weeks. Tumor volume and mouse body weight were recorded every 2 days.

NSG mice were intravenously inoculated with 1 × 10^7^ HL60-Luc cells in 0.1 mL serum-free RPMI 1640 medium on day 0. Two days after inoculation, the mice were randomly divided into four groups of 6 mice and intraperitoneally injected with vehicle, 8GL (30 mg/kg), and/or everolimus (3 mg/kg) daily for 5 continuous weeks. Leukemia burden was assessed using noninvasive bioluminescence imaging using a Xenogen IVIS-200 Optical in vivo imaging system (PerkinElmer; Waltham, MA). Bioluminescence intensity was recorded on Day 0, Day 7, and Day 14.

### Human primary AML cells

Human samples were supplied by the Department of Hematology at the 1^st^ People’s Hospital, or Xinhua Hospital, Shanghai Jiao Tong University School of Medicine. Written informed consent was obtained from the patients and approved by the Ethics Committee for Medical Research (IRB) at Shanghai Jiao Tong University School of Medicine. The patient’s information was provided in Supplemental Table S5. Human primary AML cells were maintained in StemSpan serum-free medium containing 10 ng/mL human SCF (Peprotech), 10 ng/mL human IL-3 (Peprotech), and 10 ng/mL human IL-6 (Peprotech). Human umbilical cord blood was obtained by the Translational Trials Support Laboratory at CCHMC under a protocol approved by the CCHMC Institutional Review Board. CD34^+^ cells were enriched via immunomagnetic bead selection. Cells were inoculated in IMDM with 20% calf serum supplemented with SCF, IL-3, IL-6, Flt-3L, and TPO.

GPR132 overexpression or knockdown in human primary AML cells was performed as described previously [24]. The GPR132 cDNA was cloned into a pCDH-EF1 expression vector. The specific GPR132 shRNA was cloned into pLKO.1-IRES-GFP. The sequence of shRNA was listed in Supplemental Table S4.

### Bioinformatics analyses

The dataset of AML was downloaded from TCGA (TCGA-LAML), GEO DataSets (GSE12417-GPL96, GSE12417-GPL570, GSE8970, and GSE24759), and Vizome database (BEAT-AML cohort). Gene Set Enrichment Analysis was performed using GSEA software (GSEA v4.1.0). GEPIA2 was utilized for gene expression correlation analysis [25]. PrognoScan, a database for meta-analysis of gene expression and cancer clinical prognosis, was used to analyze hazard ratios for volcano plots. Samples were grouped into lowly expressed and highly expressed groups according to the cutoff provided by PrognoScan, which gets cut off through the minimum P-value method [26]. The Kaplan-Meier plots stratified by median gene expression (*GPR132*, *ADGRL4*, *GPR37*, and *GPR6*) were performed using the GSE12417 (GEO database) or BEAT-AML cohort (http://vizome.org/aml/). Elder AML patients aged over 50 were selected for the Kaplan-Meier analysis. The significance of survival curves was calculated using a log-rank test in the GraphPad Prism 7.

### Statistical analysis

All statistical analysis was performed with unpaired Student’s *t*-test and represented as mean ± SD unless noted otherwise. The analysis of TCGA-LAML or BEAT-AML RNA-seq data for the correlation between GPR132 and AML markers was performed using a two-tailed Pearson’s correlation test. The analysis of the survival data in the PrognoScan database was performed using the log-rank (Mantel–Cox) test. The P values were designated as significant (*P < 0.05, **P < 0.01, ***P < 0.001), and non-significant (*P* > 0.05). No statistical methods were utilized to determine the sample size. Sample sizes were chosen based on previous experimental observations. The sample sizes of each experiment were shown in the figure legend. No data were excluded from the analysis. Detailed materials and methods are provided in the supplementary materials.

## Results

### Orphan G protein-coupled receptors are associated with AML prognosis

**Fig. 1:**
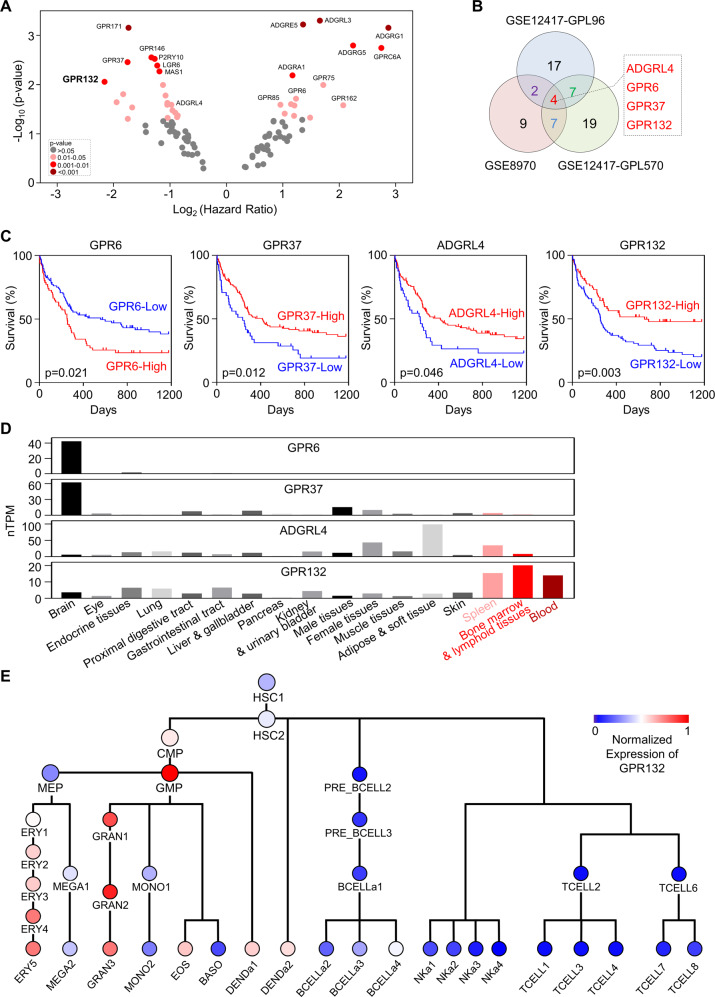
Orphan G protein-coupled receptors are associated with AML prognosis. **A** Volcano plot depicting hazard ratios (High versus low expressing group) and *P*-values of orphan GPCRs in AML patients. Data were analyzed via PrognoScan and were obtained from the GEO database: GSE12417. **B** Venn diagram representing the overlap of orphan GPCRs correlated with survival of AML patients in GSE12417-GPL96 (30 orphan GPCRs), GSE124 17-GPL570 (37 orphan GPCRs), and GSE8970 (22 orphan GPCRs). **C** Kaplan–Meier survival analysis of GPR6, GPR37, ADGRL4, and GPR132 in AML patients (GEO: GSE12417-GPL96). Based on the median gene expression level, patients were divided into high and low-expressed groups (GPR6: High/Low = 53/53; GPR37: High/Low = 53/53; ADGRL4: High/Low = 53/53; GPR132: High/Low = 53/53). Statistical significance was tested by using the log-rank test. **D** RNA expression of *GPR6*, *GPR37*, *ADGRL4*, and *GPR132* in human tissues. Data were obtained from The Human Protein Atlas (https://www.proteinatlas.org/). **E** Expression of GPR132 across the hematopoietic lineage. Data were obtained from the GEO database: GSE24759. HSC1–2: gray-hematopoietic stem cell; CMP: common myeloid progenitor; MEP: megakaryocyte/erythroid progenitor; ERY1–5: orange-erythroid cells; MEGA1–MEGA2: megakaryocyte; GMP: purple-granulocyte/monocyte progenitor; GRAN1–2: neutrophilic metamyelocyte; GRAN3: neutrophil; MONO1–2: monocytes; EOS: eosinophil; BASO: basophil; DENDa1: plasmacytoid dendritic cell; DENDa2: blue-myeloid dendritic cell; Pre_BCELL2: light green-early B-cell; Pre_BCELL3: pro-B-cell; BCELLa1: naive B-cell; BCELLa2: mature B-cell, class able to switch; BCELLa3: mature B-cell; BCELLa4: mature B-cell, class-switched; NKa1-4: dark green-mature NK cell; TCELL2: turquoise-naive CD8^+^ T-cell; TCELL1: CD8^+^ effector memory RA; TCELL3: CD8^+^ effector memory; TCELL4: CD8^+^ central memory; TCELL6: naive CD4^+^ T-cell; TCELL7: CD4^+^ effector memory; TCELL8: CD4^+^ central memory.

A list of orphan GPCRs (except olfactory receptors, Supplemental Table S1) was collected [27], and gene expression signatures of these receptors in the AML cohort were analyzed using PrognoScan [28]. We found that orphan GPCRs were differentially expressed in leukemia and exerted a pronounced clinical role in AML prognosis (Fig. 1A, Supplemental Fig. S1A, Supplemental Table S2). Of note, a subgroup of orphan GPCRs, including *ADGRL4, GPR6, GPR37*, and *GPR132*, were correlated with the survival of AML patients in three distinct gene sets of the AML cohort (Fig. 1B). Specifically, the expression levels of *GPR6* (*P* = 0.16), *GPR37* (*P* = 0.51) and *ADGRL4* (*P* = 0.51) are not associated with the prognosis of AML patients (Fig. 1C). In contrast, the increased expression level of *GPR132* (*P* = 0.014) predicted a superior prognosis, suggesting a potential protective role of GPR132 in AML (Fig. 1C, Supplemental Fig. S1B, Supplemental Table S3). AML blasts exhibit a lower expression level of *GPR132* than normal bone marrow mononuclear cells from healthy donors (BEAT-AML cohort) [29] (Supplemental Fig. S1C). Additionally, tissue-specific expression analysis using The Human Protein Atlas showed that GPR132 is predominantly enriched in bone marrow (Fig. 1D) [30, 31].

We further determined the expression level of GPR132 across normal hematopoiesis development using a publicly available dataset [32]. We found that GPR132 expression was enriched in the myeloid lineage during normal hematopoietic differentiation, but not hematopoietic stem cells (HSCs) (Fig. 1E), suggesting a myeloid-specific expression of GPR132 that has minor or no function in HSCs activities. Considering the expression patterns and disease correlations, we focused on the potential role of GPR132 in leukemogenesis.

### Orphan receptor GPR132 triggers myeloid differentiation in AML

**Fig. 2:**
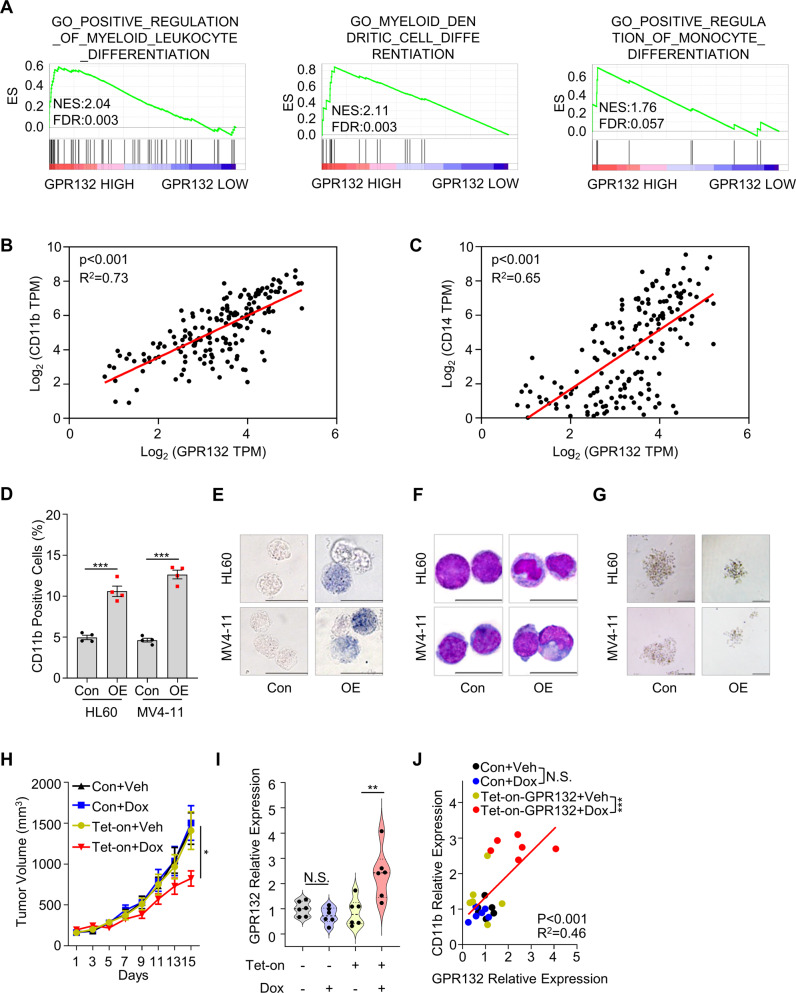
Orphan G protein-coupled receptor GPR132 triggers myeloid differentiation in AML. **A** Gene set enrichment analysis (GSEA) of the TCGA AML dataset comparing GPR132 HIGH and GPR132 LOW. Gene set involving positive regulation of myeloid leukocyte differentiation is shown. ES, enrichment score; NES, normalized enrichment score; FDR, false discovery rate. **B**, **C** Gene expression correlation between *GPR132* and *CD11b* (**B**) or *CD14* (**C**) in TCGA-LAML dataset. A two-sided Pearson’s correlation analysis was performed. **D** Flow-cytometric analysis of myeloid differentiation marker CD11b expression using Tet-On AML cells (HL60 and MV4-11) with (OE) or without (Con) doxycycline treatment (1 μg/mL) for 72 h. Data are shown as means ± SEM (*n* = 3). Student’s *t*-tests were performed, ***P < 0.001. **E** NBT reduction analysis showing formazan formation in Tet-On AML cells (HL60 and MV4-11) with or without doxycycline exposure (1 μg/mL) for 72 h. Scale bars represent 20 μm. **F** Representative Wright–Giemsa staining images showing the mature myeloid cell morphology of Tet-On AML cells (HL60 and MV4-11) following doxycycline treatment (1 μg/mL, 72 h). Scale bars represent 20 μm. **G** Representative images of colony formation for Tet-On AML cells. Cells were cultured in a semi-solid medium and treated with 1 μg/mL doxycycline to induce GPR132 overexpression. Colonies were photographed under a microscope on day 7. Scale bars represent 100 μm. **H** GPR132 delays growth and promotes differentiation of AML in vivo. GPR132 Tet-On HL60 cells were injected into the mid-right flank of female athymic nude mice subcutaneously. When tumor volumes reached nearly 200 mm^3^, mice were treated with doxycycline (1 mg/mL in drinking water) for 2 weeks. Tet-On represents Tet-On-GPR132. Data are presented as means ± SEM (*n* = 6). Student’s *t*-tests were performed, *P < 0.05. **I** Expression analysis of *GPR132* determined using qPCR in Tet-On HL60 xenograft mice model. Tet-On represents Tet-On-GPR132. Student’s *t*-tests were performed (*n* = 6), N.S., not significant, **P < 0.01. **J** Correlation analysis of GPR132 and CD11b expression in tumor tissues. **H** Expression levels were detected using qPCR. Student’s *t*-tests were performed (*n* = 6), N.S., not significant, ***P < 0.001.

To explore the role of GPR132 in AML, we performed gene set enrichment analysis (GSEA) using The Cancer Genome Atlas (TCGA) AML dataset [33]. Intriguingly, we observed that myeloid leukocyte differentiation was the most enriched biological pathway associated with GPR132 expression (Fig. 2A, Supplemental Fig. S2A). Consistently, gene co-expression analyses in both of the TCGA AML dataset [33] and the Vizome BEAT-AML dataset [29] confirmed the positive correlation between GPR132 and two well-known myeloid differentiation markers, CD11b and CD14 (Fig. 2B, C, Supplemental Fig. S2B, C) [34, 35]. These results indicated that GPR132 may be involved in the promotion of AML cell differentiation.

Next, we found that GPR132 was widely expressed in different subtypes of AML cell lines of French–American–British (FAB) classification (Supplemental Fig. S2D, E), including Kasumi-1 (M2), HL60 (M2), NB4 (M3), OCI-AML3 (M4), MV4–11 (M5), THP-1 (M5), and MOLM13 (M5) cells. AML FAB-M2 was responsive to differentiation therapy and AML FAB-M5, one of the most severe leukemia types, was largely resistant to clinical treatment [36, 37]. Therefore, we chose two FAB subtypes of the AML cell lines mentioned above, HL60 (M2) and MV4–11 (M5), for exogenous GPR132 expression using a Tet-based inducible expression system to determine its function in leukemogenic activities. We observed that treatment with doxycycline effectively induced GPR132 overexpression in both HL60 (Tet-On-GPR132) and MV4–11 (Tet-On-GPR132) cells (Supplemental Fig. S2F, G). Subsequently, the differentiation of AML cells was assessed. Flow cytometric analysis showed that GPR132 overexpression significantly upregulated the percentage of CD11b^+^ in both HL60 (Tet-On-GPR132) and MV4–11 (Tet-On-GPR132) cells (Fig. 2D and Supplemental Fig. S2H). SPI-1 and C/EBPα were two known transcription factors for up-regulating the expression of CD11b and CD14, and promoting myeloid cell differentiation [38–40]. We found that overexpression of GPR132 elevated the mRNA levels of *SPI1* and *CEBPA* (Supplemental Fig. S2I, J). Secondly, nitro-blue tetrazolium (NBT) reduction, a reaction indicating myeloid differentiation [41, 42], was enhanced by GPR132 overexpression in AML cells (Fig. 2E, Supplemental Fig. 2K). Finally, Wright–Giemsa staining-based morphological analysis [19, 43] demonstrated that AML cells with exogenous GPR132 exhibit an increased cytoplasmic-to-nuclear ratio with nuclear segmentation or bending (Fig. 2F, Supplemental Fig. 2L). In addition, we found a significant reduction in the number of CFU in the GPR132-overexpressing AML cell lines (Fig. 2G, Supplemental Fig. S2M, N) in colony formation analysis [24, 44]. Doxycycline alone did not affect cell differentiation and colony-forming ability of AML cells (Supplemental Fig. S2O-Q).

To further verify the function of GPR132 in vivo, a subcutaneous xenograft model was established using HL60 (Tet-On-GPR132) cells. Notably, we found that GPR132 overexpression following doxycycline treatment significantly impaired the growth of HL60 (Tet-On-GPR132) xenograft (Fig. 2H). However, the effect of doxycycline was lost in parental HL60 tumors (Fig. 2H). Doxycycline treatment exerted no effect on the body weights of mice (Supplemental Fig. S2R). Furthermore, consistent with the in vitro results, GPR132 overexpression boosted the levels of CD11b in tumor tissues (Fig. 2I, J). Together, our data indicated that the orphan receptor GPR132 triggers cell differentiation and induces cell growth inhibition of AML cells.

### A natural product, 8-gingerol, is an agonist of GPR132

**Fig. 3:**
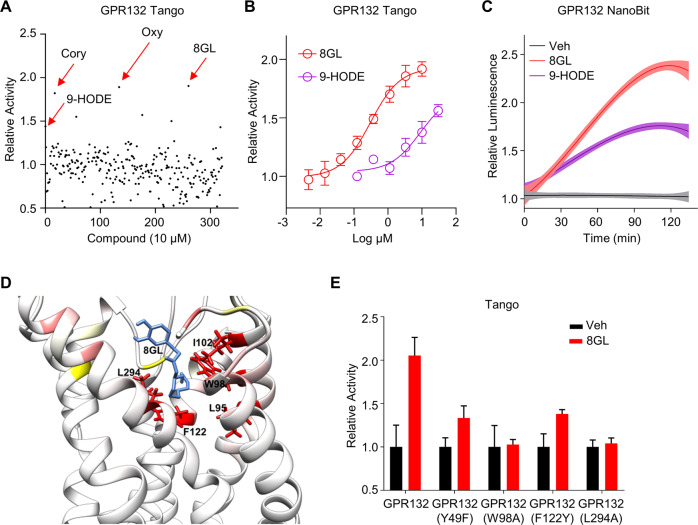
Screening and validation of 8GL as a GPR132 agonist. **A** Dot plot showing screening results of 322 compounds evaluated with a concentration of 10 μM in a GPR132 Tango assay. Each dot represents one compound. The top three hits were 8-Gingerol (8GL), oxyresveratrol (Oxy), and corynoline (Cory). **B** Dose–response curve of 8GL and 9-HODE for GPR132 activation (EC_50_ = 0.30 μM) in Tango assays. Data are shown as means ± SEM (*n* = 3). **C** Kinetic curves of 8GL (10 μM) or 9-HODE (10 μM) in GPR132-NanoBiT assays using GPR132-HEK293 cells. Luminescence signals were measured at 40-s intervals. **D** Predicted binding mode involving 8GL and GPR132. **E** Agonistic activity of 8GL for wild-type GPR132 and mutant GPR132 in Tango assays.

Next, we sought to explore new GPR132 agonizts to probe the therapeutic role of GPR132 activation in AML. Several endogenous lipid molecules were reported to activate GPR132 [45], whereas the low potency and specificity largely limited their therapeutic applications. An in-house phytochemical library was screened for its agonistic activities on GPR132 using a GPCR-Tango assay (Supplemental Fig. S3A) [46]. 9-hydroxy-10E,12Z-octadecadienoic acid (9-HODE), a putative endogenous agonist of GPR132 [45], was used as a control compound with a half-maximal effective concentration (EC_50_) value of 9.00 μM (Fig. 3B). Preliminary screening of compounds at 10 μM demonstrated that 8-gingerol (8GL), corynoline (Cory), and oxyresveratrol (Oxy) emerged as the most potent GPR132 agonizts (Fig. 3A, Supplemental Fig. S3B). Among these compounds, 8GL is a selective agonist of GPR132 without promiscuous activity against a panel of receptors at 10 μM (Supplemental Fig. S3C). Notably, 8GL exhibited superior GPR132 agonistic activity than 9-HODE and had an EC_50_ value of 0.30 μM (Fig. 3B).

We further performed multiple functional GPCR assays. 8GL treatment resulted in a 2.4-fold increase in luminescence signal in β-arrestin-based NanoBit assays, which is more potent than 9-HODE at the same concentration (10 μM) (Fig. 3C). Moreover, 8GL demonstrated potent GPR132 agonistic activity in CRE luciferase assays [47], with an EC_50_ value of 0.44 μM (Supplemental Fig. S3D), consistent with the results of Tango assays.

Molecular docking revealed that 8GL interacted with residues Y49, W98, F122, and L294 of GPR132 (Fig. 3D). Mutations of these residues resulted in a lower response of GPR132 to 8GL in Tango assays, indicating that these residues are essential for 8GL binding of GPR132 (Fig. 3E). The membrane anchoring of GPR132 were not influenced by these mutations (Supplemental Fig. S3E). Collectively, we concluded that 8GL represents an agonist of the orphan receptor, GPR132.

### Activation of GPR132 by 8GL triggers AML cell differentiation

**Fig. 4:**
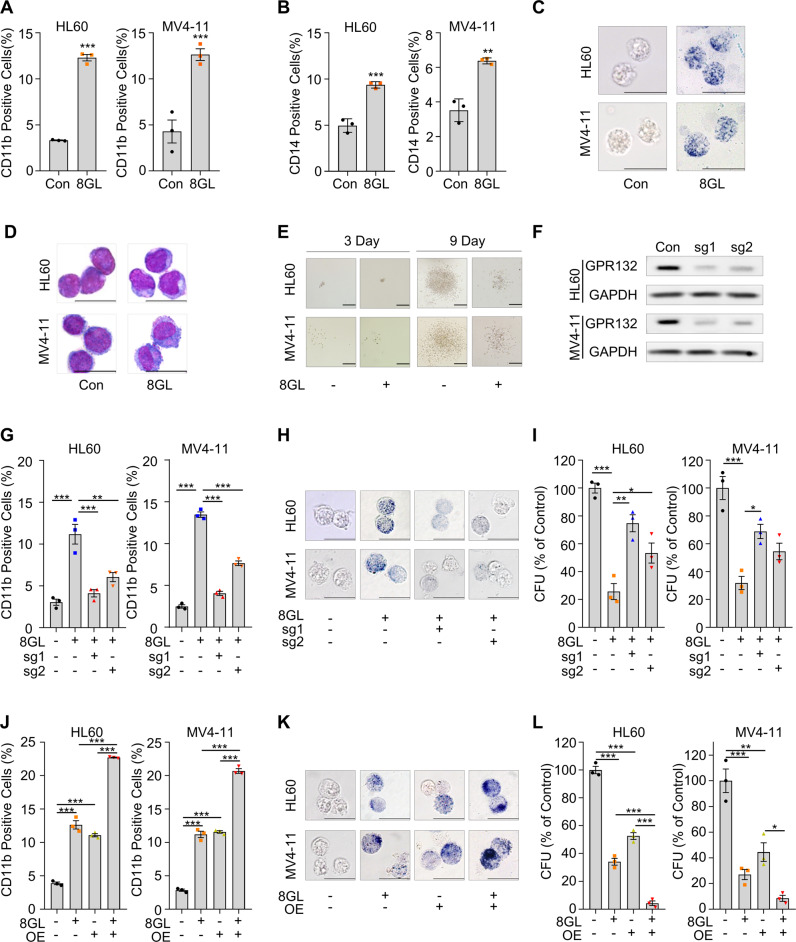
8GL induces the differentiation and suppresses the proliferation of AML cells via GPR132. **A**, **B** Flow cytometric analysis of cell surface markers CD11b (**A**) and CD14 (**B**) in HL60 and MV4-11 cells 72 h after 8GL treatment (30 μM). One-way analysis of variance (ANOVA) with Tukey’s multiple comparison tests was performed (*n* = 3), **P < 0.01, ***P < 0.001. **C** NBT reduction analysis demonstrating formazan generation in AML cell lines HL60 and MV4-11. HL60 and MV4-11 cells were treated with 8GL (30 μM) for 72 h. Scale bars represent 20 μm. **D** Representative Wright–Giemsa staining results showing the mature myeloid cell morphology of HL60 and MV4-11 cells treated with 8GL (30 μM) for a period of 72 h. Scale bars represent 10 μm. **E** Representative results of colony formation assays involving HL60 and MV4-11 cells. AML cells were cultured in a semi-solid medium and incubated with 8GL (15 μM). Colonies were photographed under a microscope on days 3 and 9. Scale bars represent 100 μm. **F** Western blotting analysis of GPR132 expression in GPR132-knockout HL60 and MV4-11 cell lines. Sg1 and sg2 represent two distinct sgRNAs for *GPR132*. An anti-GPR132 antibody was purchased from Santa Cruz (sc-137112). Anti-GAPDH antibody was purchased from Cell Signaling Technology (5174). **G** Flow cytometric analysis of CD11b in 8GL-treated GPR132-WT and GPR132-KO AML cell lines. 8GL treatment: 30 μM for 72 h. Data are presented as mean ± SEM (*n* = 3). Sg1 and sg2 represent two distinct sgRNAs for *GPR132*. One-way ANOVA with Tukey’s multiple comparison tests was performed, **P < 0.01, ***P < 0.001. **H** Representative NBT reduction images showing formazan formation in GPR132 wild-type (WT) and GPR132 knockout (KO) AML cell lines. GPR132-WT and GPR132-KO cells were treated with 8GL at a concentration of 30 μM for 72 h. Scale bars represent 20 μm. Sg1 and sg2 represent two distinct sgRNAs for *GPR132*. **I** Colony formation in GPR132-WT and GPR132-KO AML cell lines in the presence of 8GL. 8GL treatment: 15 μM for 7 days. Sg1 and sg2 represent two distinct sgRNAs for *GPR132*. One-way ANOVA with Tukey’s multiple comparison tests was performed (*n* = 3), *P < 0.05, **P < 0.01, ***P < 0.001. **J**, **K** Tet-On AML cells were treated with 8GL (30 μM for 72 h) in the presence or absence of doxycycline (1 μg/mL), and myeloid differentiation was detected based on CD11b expression analysis using flow cytometry (**J**) and NBT reduction analysis (**K**). The scale bar represents 10 μm. Data are presented as mean ± SEM (*n* = 3). One-way ANOVA with Tukey’s multiple comparison tests was performed, ***P < 0.001. **L** Tet-On AML cells were incubated with 8GL (15 μM for 7 days) in the presence or absence of doxycycline (1 μg/mL), and cell growth was determined by colony formation analysis. Data are presented as mean ± SEM (*n* = 3). One-way ANOVA with Tukey’s multiple comparison tests was performed, *P < 0.05, **P < 0.01, ***P < 0.001.

Next, we evaluated the effects of GPR132 activation by 8GL on AML cell differentiation. Treatment with 8GL induced elevation of protein levels of myeloid differentiation markers of CD11b and CD14 and upregulation of mRNA levels of transcription factors of *SPI1* and *CEBPA* in HL60 and MV4–11 cells (Fig. 4A, B, Supplemental Fig. S4A–D). NBT reduction and Wright–Giemsa staining also indicated enhanced differentiation in the 8GL-treated HL60 and MV4–11 cells (Fig. 4C, D). 8GL also impaired the colony-forming ability of these two AML cell lines in methylcellulose-based 3D culture (Fig. 4E, Supplemental Fig. S4E). In addition to HL60 (with mutation of *NRAS*, *M2*) and MV4–11 (with mutation of *FLT3*, *M5*), 8GL could induce differentiation in other AML cell lines, such as OCI-AML3 (with mutation of *DNMT3A, M4*) and THP1 (with mutation of *CDKN2A, M5*) (Supplemental Fig. S4F, G). Additionally, 8GL inhibited the proliferation of HL60 and MV411 cells with minor cytotoxicity (Supplemental Fig. S4H, I). And 8GL has no effect on both cell growth and cell viability of the healthy human HSCs (Supplemental Fig. S4J). Together, these results confirmed the differentiation-inducing activity of 8GL in AML with disparate genetic profiles and FAB subtypes.

We then queried whether GPR132 was responsible for the 8GL pro-differentiation capacity. To this end, we established *GPR132* knockout cells using CRISPR-Cas9 (Fig. 4F) [15]. Notably, the GPR132 knockout diminished the effectiveness of 8GL. Specifically, 8GL treatment failed to induce upregulation of CD11b expression, deposition of NBT residue, and alteration of cytoplasmic-to-nuclear ratio (Fig. 4G, H, Supplemental Fig. S4K). Additionally, the reduced colony formation of HL60 and MV4–11 by 8GL was recovered by sgRNA-mediated *GPR132* knockout (Fig. 4I and Supplemental Fig. S4L).

In contrast, exogenous GPR132 expression enhanced 8GL-induced myeloid differentiation. The expression levels of CD11b and CD14 and NBT reduction were synergistically augmented by GPR132 overexpression and 8GL treatment (Fig. 4J, K, Supplemental Fig. S4M, N). In colony formation assays, exogenous GPR132 expression and 8GL treatment had comparable inhibitory activities, whereas their combination nearly completely abolished AML cell growth (Fig. 4L and Supplemental Fig. S4O). In summary, these findings suggested that 8GL is a chemical inducer for myeloid differentiation by activating GPR132.

### 8GL disrupts mTOR signaling in AML cells by activating the GPR132-PKA pathway

**Fig. 5:**
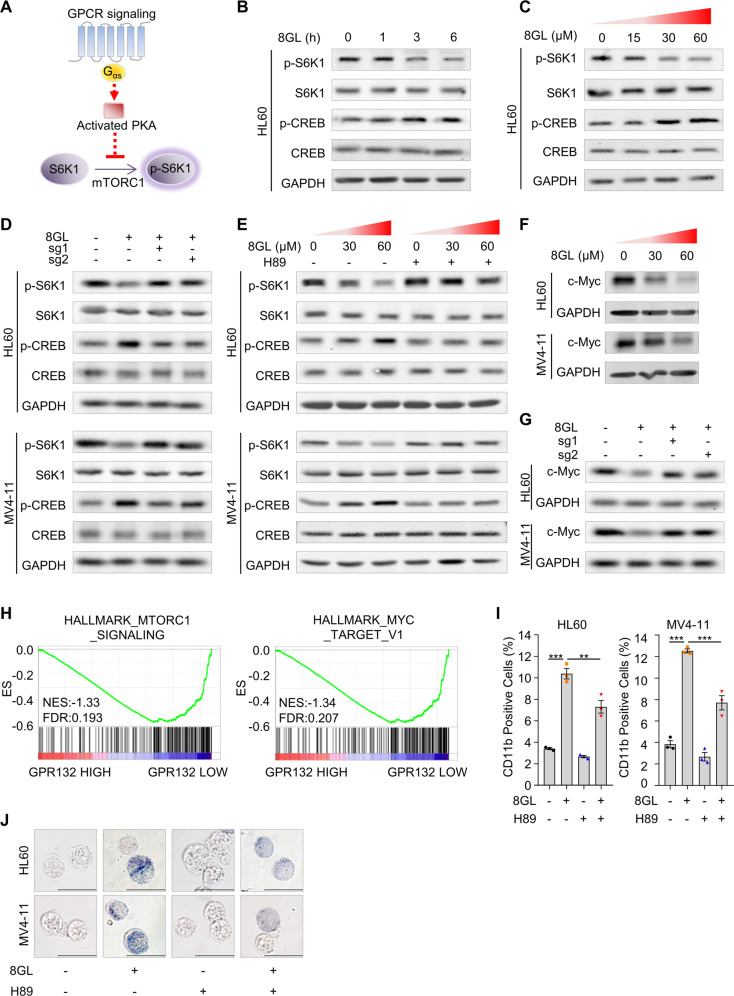
8GL represses mTORC1 activity via the GPR132-PKA pathway in AML. **A** Schematic illustration of the GPCR-Gs-PKA signaling axis that inhibits mTORC1. **B** HL60 cells were treated with 8GL (30 μM) for indicated times. The levels of p-S6K1 (Thr389) and p-CREB (Ser133) were measured using western blotting. **C** HL60 cells were treated with 8GL at concentrations of 0, 15, 30, and 60 μM. The levels of p-S6K1 (Thr389) and p-CREB (Ser133) were measured using western blotting. The antibodies were purchased from Cell Signaling Technology (CST). p-S6K1, CST-9205; S6K1, CST-9202; p-CREB, CST-9198; CREB, CST-9197. **D** Western blotting analysis of p-S6K1 (Thr389) and p-CREB (Ser133) in 8GL-treated GPR132 WT and GPR132 KO AML cell lines. 8GL treatment: 30 μM for 3 h. Sg1 and sg2 represent two distinct sgRNAs for *GPR132*. **E** Phosphorylation status of S6K1 (Thr389) and CREB (Ser133) in HL60 and MV4-11 cells incubated with 8GL (30 μM or 60 μM) and/or H89 (1 μM). **F** HL60 and MV4-11 cells were treated with 8GL (0 μM, 30 μM, and 60 μM) for 48 h, and c-Myc expression was detected using western blotting. **G** Western blot analysis of c-Myc protein in GPR132 WT and GPR132 KO AML cells in the presence of 8GL (30 μM for 48 h). Sg1 and sg2 represent two distinct sgRNAs for *GPR132*. **H** Gene set enrichment analysis of the GSE12417-GPL96 dataset comparing GPR132 HIGH (top 10% of GPR132 expression) and GPR132 LOW (top 10% of GPR132 expression). **I** HL60 and MV4-11 cells were treated with 8GL (30 μM) and/or H89 (1 μM) for 72 h, and CD11b expression was detected using flow cytometry. Data are shown as mean ± SEM (*n* = 3). One-way ANOVA with Tukey’s multiple comparison tests was performed. **P < 0.01, ***P < 0.001. **J** Representative NBT reduction results of AML cell lines treated with or without 30 μM 8GL and/or 1 μM H89 for 72 h. The scale bar represents 10 μm.

Next, we investigated the underlying mechanism by which 8GL triggered the differentiation of AML cells. The mTOR signaling pathway plays a prominent role in the regulation of differentiation blockade and clonal expansion of leukemic cells [48, 49]. Recently, the Guan group [50] reported that the G_αs_-cAMP-PKA axis is involved in the negative regulation of mTOR signaling in non-cancerous HEK293A cells (Fig. 5A). We thus hypothesized that GPR132 activation by 8GL may downregulate mTOR activity by stimulating the G_αs_-cAMP-PKA pathway in leukemia [51]. To test this, forskolin (FSK), an activator of cAMP generation [52], was used as a positive control for G_αs_ signaling. Activation of PKA was detected via phosphorylation of CREB at Ser 133 [53], a direct target of PKA, and the activity of mTOR was measured by the phosphorylation of S6K1, a well-characterized mTOR substrate [54]. FSK significantly increased the level of p-CREB, accompanied by a decreased level of p-S6K1, in a concentration- and time-dependent manner in HL60 cells (Supplemental Fig. S5A, B). Nevertheless, the reciprocal effects of FSK on the phosphorylation of S6K1 and CREB were abrogated by the PKA inhibitor H89 (Supplemental Fig. S5C, D). These results confirmed the negative regulation of mTOR activity by the GPR132-PKA axis in the context of AML.

Treatment with 8GL markedly elevated the level of p-CREB, but largely diminished the expression of p-S6K1 in HL60 cells (Fig. 5B, C, Supplemental Fig. S5E, F). In contrast, the effect of 8GL on p-CREB and p-S6K1 was nearly completely abolished in GPR132-knockout AML cells (HL60 and MV4–11) (Fig. 5D, Supplemental Fig. S5G, H). Similar results were also observed following treatment with the PKA inhibitor H89 (Fig. 5E, Supplemental Fig. S6A, B), indicating a PKA-dependent regulatory mechanism involving mTOR. c-Myc is a pivotal downstream transcription factor of mTOR signaling in the regulation of cell proliferation and differentiation [55, 56]. The protein levels of c-Myc were significantly downregulated by 8GL (Fig. 5F, Supplemental Fig. S6C). Nevertheless, the c-Myc reduction was almost fully rescued by *GPR132* deletion or H89 (Fig. 5G, Supplemental Fig. S6D, E). Consistent with these observations, GSEA analysis [57] revealed enriched profiles of mTORC1 signaling and Myc in patients with low GPR132 expression, rather than in patients with high GPR132 levels (Fig. 5H). Taken together, we concluded that GPR132 activation by 8GL suppresses mTOR signaling in AML cells via the PKA pathway.

Next, we ascertained the functional contribution of GPR132-PKA signaling in myeloid differentiation. PKA inhibition by H89 largely impaired 8GL-elicited upregulation of the differentiation markers CD11b and CD14 (Fig. 5I, Supplemental Fig. S6F–H). In addition, increased NBT reduction and mature morphological properties induced by 8GL were negated by H89 (Figs. 5J, S6I). Correspondingly, reduced colony formation in AML cells was recovered in the presence of H89 (Supplemental Fig. S6J, K). In summary, these results demonstrated that 8GL triggers AML cell differentiation by activating the GPR132-PKA pathway, which disrupts mTOR signaling.

### Synergistic antileukemic effects of the 8GL and mTOR inhibitor everolimus combination in vitro

**Fig. 6:**
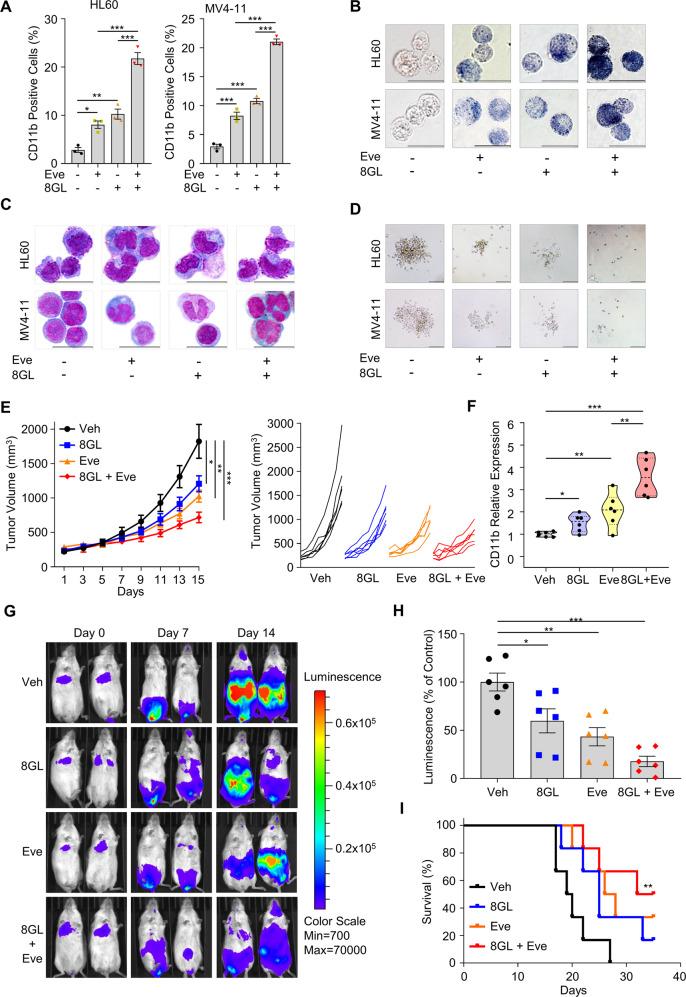
Drug combination of 8GL and a mTORC1 inhibitor shows synergistic antileukemic effects in vitro and in vivo. **A** HL60 and MV4-11 cells were treated with vehicle, 30 μM 8GL, 3 μM everolimus (Eve), or drug combination for 72 h, and the expression of CD11b was determined using flow cytometry. Data are shown as mean ± SEM (*n* = 3). One-way ANOVA with Tukey’s multiple comparison tests was performed, *P < 0.05, **P < 0.01, ***P < 0.001. **B** Representative images of NBT reduction in HL60 and MV4-11 cells upon co-treatment of 8GL and Eve. Cells were treated as described in (**A**). Scale bars represent 20 μm. **C** Representative Wright–Giemsa staining images showing the mature myeloid cell morphology of HL60 and MV4-11 cells induced by 8GL and Eve combination. Cells were treated as described in (**A**). Scale bars represent 20 μm. **D** Representative images of colony formation of HL60 and MV4-11 cells incubated with 8GL (15 μM) and/or Eve (1.5 μM) for 7 days. The scale bar represents 100 μm. **E** Tumor growth curves of BALB/c nude mice inoculated with 1 × 10^6^ HL60 cells. Mice were administrated with 8GL (30 mg/kg/d) in combination with Eve (3 mg/kg/d). Data are presented as mean ± SEM (*n* = 6). One-way ANOVA with Tukey’s multiple comparison tests was performed, *P < 0.05, **P < 0.01, ***P < 0.001. **F** Expression analysis of CD11b was determined using qPCR in the HL60 xenograft model as indicated in (**E**). Student’s *t*-tests were performed (*n* = 6), *P < 0.05, **P < 0.01, ***P < 0.001. **G** Bioluminescent imaging of NOD.Cg-Prkdc^scid^Il2rg^tm1Wjl^/SzJ (NSG) mice transplanted with HL60-Luc cells treated with vehicle, 8GL (30 mg/kg), and/or everolimus (3 mg/kg). Representative images of two mice per group are shown. **H** Bioluminescence quantification representing tumor mass (count) on days post-injection of HL60-Luc cells. Mean normalized values are plotted for each treatment group. Data are presented as mean ± SEM (*n* = 6). Student’s *t*-tests were performed, *P < 0.05, **P < 0.01. **I** Kaplan–Meier overall survival plot of HL60-Luc xenograft AML mice model following treatment with vehicle, 8GL (30 mg/kg), and/or Eve (3 mg/kg) (*n* = 6). Statistical significance was tested using the log-rank test, *P < 0.05, **P < 0.01.

Considering the poor clinical efficacy of a single treatment with mTOR inhibitors [58, 59], we then investigated whether 8GL could potentiate the anticancer efficacy of everolimus via the co-inhibition of the mTOR pathway in AML. Notably, dual treatment with 8GL and everolimus produced a much higher expression of CD11b and CD14 when compared to either single drug alone (Fig. 6A, Supplemental Fig. S7A–C). Similar synergetic results were observed in terms of the detection of other differentiation features, including NBT reduction, Wright–Giemsa staining, and colony formation (Fig. 6B–D, Supplemental Fig. S7D). Correspondingly, 8GL dramatically strengthened the blocking of mTOR signaling by everolimus, as reflected by the largely reduced p-S6K levels (Supplemental Fig. S7E).

Furthermore, the combination of 8GL and everolimus inhibited cell viability more efficiently than either of these monotherapies alone (Supplemental Fig. S7F). To assess the synergistic effects of 8GL and everolimus, we quantified the drug combination index (CI) using the CalcuSyn software (Version 2; Biosoft) [18]. In HL60 and MV4–11 cell lines, the CI values of 8GL and everolimus were both <1, suggesting a synergistic effect (Supplemental Fig. S7F). We also found that GPR132 activation by 8GL-sensitized chemotherapeutic drugs, including doxorubicin, azacytidine, and decitabine (Supplemental Fig. S7G–I). Taken together, these results suggested that 8GL and everolimus cooperatively boosted myeloid differentiation by co-targeting mTOR signaling, offering a potential drug combination approach with superior therapeutic efficacy.

### 8GL alone and in combination with everolimus exhibits in vivo activity in murine AML model

To determine the in vivo effects of 8GL on AML, we established an HL60 xenograft model in mice. Mice receiving 8GL demonstrated a marked reduction in in vivo tumor growth (35.89 ± 8.69% and 54.12 ± 9.44%, 30 and 60 mg/kg 8GL, respectively), compared to the control group (Supplemental Fig. S8A). Similarly, the weights of tumors in the 8GL-treated groups were markedly decreased (Supplemental Fig. S8B). Notably, a strong elevation of the differentiation marker CD11b indicated in vivo cell differentiation upon 8GL treatment (Supplemental Fig. S8C). Specifically, CD11b levels in the high-dose group were almost fourfold that of the control group, and its expression was negatively correlated with tumor weight (Supplemental Fig. S8C). No significant change in body weight was observed, suggesting low toxicity of 8GL treatment (Supplemental Fig. S8D). These findings indicate the profound efficacy of 8GL in triggering cell differentiation and in impairing cell proliferation in a murine leukemia xenograft model in vivo.

We then examined the in vivo potency of the drug combination of 8GL and everolimus. Compared with the control group, monotherapy with 8GL or everolimus resulted in moderate tumor growth inhibition (36.92 ± 6.10% for 8GL, 46.27 ± 4.58% for everolimus), whereas the drug regimen involving 8GL and everolimus significantly impaired tumor growth (62.50 ± 4.03% for drug pair) (Fig. 6E). Similar results were obtained for tumor weight at the end of treatment (Supplemental Fig. S8E). Co-administration of 8GL and everolimus resulted in CD11b upregulation, compared to the control group, suggesting an effective in vivo differentiation-inducing capacity (Fig. 6F). The combination therapy is well tolerated and has no side effects at the given dosages (Supplemental Fig. S8F).

To further examine the activity of the combined therapy in vivo, we generated a systemic leukemia model by intravenous injection of HL60-Luc cells. Tumors in the vehicle-treated group grew rapidly and diffused throughout the mouse body by day 14. A single treatment with 8GL or everolimus retarded tumor growth, whereas the combination therapy showed superior anti-tumor activity than either of the single drug used alone (Fig. 6G, H). The survival of tumor-bearing mice has largely improved: the vehicle-treated group (median survival, 19.5 days), 8GL-treated group (median survival, 25.0 days), everolimus-treated group (median survival, 27.0 days), and the drug combination group (median survival, 33.5 days) (Fig. 6I). Taken together, combination therapy with 8GL and an mTOR inhibitor effectively inhibited tumor growth and prolonged mouse survival.

### GPR132 enhances the differentiation of human primary AML cells

**Fig. 7:**
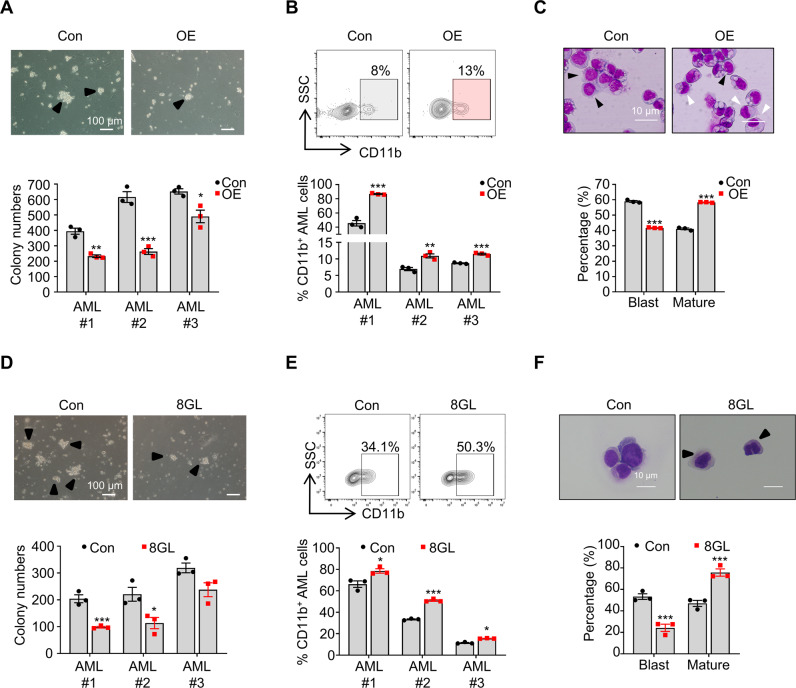
GPR132 enhances human primary AML cell differentiation. **A** Representative images (upper panel) and quantitative data (lower panel) for colony numbers of GPR132-overexpressing (OE) or control (Con, empty vector) human primary AML cells. Data are presented as mean ± SEM (*n* = 3). Student’s *t*-tests were performed, *P < 0.05, **P < 0.01, ***P < 0.001. The scale bar represents 100 μm. **B** Representative contour plots (upper panel) and quantitative data (lower panel) of flow cytometric analysis for the percentages of CD11b^+^ cells in GPR132-overexpressing (OE) or control (Con, empty vector) human primary AML cells. Data are presented as mean ± SEM (*n* = 3). Student’s *t*-tests were performed, **P < 0.01, ***P < 0.001. **C** Representative images of Wright–Giemsa staining (upper panel) and quantitative data of the percentage of blast cells (black arrowhead) and differentiated cells (mature cells, white arrowhead, lower panel) of GPR132-overexpressing (OE) or control (Con) human primary AML cells. A total of 20‒30 cells were counted for each section, and 8‒10 sections were evaluated. Data are presented as mean ± SEM (*n* = 3). Student’s *t*-tests were performed, ***P < 0.001. **D** Representative images (upper panel) and quantitative data (lower panel) of colony numbers of human primary AML cells after treatment with vehicle (0 μM DMSO) or 8GL (30 μM). Data are presented as mean ± SEM (*n* = 3). One-way ANOVA with Tukey’s multiple comparison tests was performed, *P < 0.05, ***P < 0.001. **E** Representative contour plots (upper panel) and quantitative data (lower panel) of flow cytometric analysis for the percentage of CD11b^+^ cells in human primary AML cells after treatment with vehicle (0 μM DMSO) or 8GL (30 μM). Data are presented as mean ± SEM (*n* = 3). One-way ANOVA with Tukey’s multiple comparison tests was performed, *P < 0.05, ***P < 0.001. **F** Representative images of Wright–Giemsa staining (upper panel) and quantification data of percentages of blast cells (black arrowhead) and differentiated cells (mature cells, white arrowhead, lower panel) of human AML cells after treatment with vehicle (0 μM DMSO) or 8GL (30 μM). A total of 20‒30 cells were counted for each section, and 8–10 sections were evaluated. Data are presented as mean ± SEM (*n* = 3). One-way ANOVA with Tukey’s multiple comparison tests was performed, ***P < 0.001.

Next, we investigated whether GPR132 could enhance the differentiation of human primary AML cells. We initially overexpressed GPR132 in human primary AML cells and demonstrated that GPR132-overexpressing cells had ~25‒57% fewer colony numbers than control cells, as determined by an in vitro colony forming assay (Fig. 7A). Overexpression of GPR132 in human primary AML cells led to a significant increase in the expression level of CD11b, a surface marker for human leukemia cell differentiation (Fig. 7B). Consistently, more differentiated mature AML cells were observed upon GPR132 overexpression, as evaluated by Wright–Giemsa staining (Fig. 7C). We then further constructed shRNA to downregulate GPR132 levels in human primary AML cells and revealed that GPR132-knockdown cells could generate ~1.7‒2.4-fold more colonies than scramble-shRNA-treated AML cells (Supplemental Fig. S9A-C). GPR132-knockdown AML cells had much lower expression levels of CD11b (Supplemental Fig. S9D, E), which was consistent with the increased number of undifferentiated blast cells upon GPR132 knockdown as evaluated by Wright-Giemsa staining (Supplemental Fig. S9F, G). Taken together, these data indicated that the presence of GPR132 promoted the differentiation of blast cells derived from AML patients.

Finally, we examined the effect of the GRP132 agonist 8GL on human primary AML cells and found that 8GL could effectively inhibit colony generation (Fig. 7D). Treatment with 8GL also significantly increased the frequency of CD11b^+^ differentiated AML cells (Fig. 7E), and a decrease in blast cell number (Fig. 7F), indicating that the activation of GPR132 by agonizts (such as 8GL) may serve as a potent therapeutic strategy for the treatment of AML.

## Discussion

Herein, we described an integrated bioinformatic and experimental approach that identified orphan receptor GPR132 as a differentiation trigger in AML, with distinct genetic alteration profiles and FAB subtypes. To the best of our knowledge, this is the first study to reveal the role of orphan GPR132 differentiation induction and its underlying mechanism of action in hematological cancer. Our work underlines the advantage of combining functional genomics and experimental validation, providing new drug targets and biological insights.

Numerous members of the GPCR family have been exploited as druggable targets in medicine, though >100 non-olfactory orphan GPCRs remain poorly characterized [10]. GPR132 represents one such example. GPR132 is mainly expressed in immune cells, including macrophages, B cells, and T cells [60, 61]. Several previous studies have shown that GPR132 is involved in the regulation of cell migration, polarization, activation of macrophages, [62–64], and hematopoiesis [65]. Herein, we have demonstrated a functional role for an orphan receptor, GPR132, in modulating abnormal myeloid differentiation, as evidenced by using both cell lines and human primary blast cells of AML patients. The differentiation-inducing function of GPR132 in leukemic blast cells establishes a corroborative link between GPCR signaling and cell differentiation decisions. Further investigations were warranted to define the role of GPR132 in cell cycle, cell apoptosis, and cell migration of leukemia blasts.

Several endogenous oxygenated free fatty acids, including 9(S)-HODE, have been reported to be potential ligands of GPR132 with micromolar potency (9(S)-HODE: EC_50_ = 9 μM) [45, 65]. However, considering the moderate potency and limited physiological concentrations in tissues, levels of these unstable lipid molecules may be too low to effectively simulate GPR132 in vivo. Correspondingly, none of these lipid molecules were tested as GPR132 agonizts in animal models. Formally, GPR132 has been recognized as an orphan receptor [27]. Thus, the discovery of new agonizts would profoundly facilitate the characterization of the biological roles and therapeutic potential of orphan receptor GPR132.

In this study, natural 8GL was identified as a GPR132 agonist. We further observed that 8GL impaired leukemic cell growth and extended mouse survival in preclinical AML models, likely through direct activation of orphan receptor GPR132. Notably, 8GL effectively triggered the differentiation of human primary blast cells derived from AML patients. These comprehensive preclinical results demonstrated 8GL’s anti-leukemic activity as a GPR132 agonist. Considering the chemical skeleton differences distinguishing 8GL and other reported in vitro GPR132 agonizts, as well as it is in vivo safety and efficiency in AML models, our results support a rationale for exploiting more potent and specific GPR132-targeted 8GL derivatives for the treatment of different types of AML. A recent study has demonstrated that EGFR/STAT/ERK pathway is involved in the anti-cancer activities of 8GL in colorectal cancer in vitro [51]. Thus, further studies are needed to investigate whether this pathway is implicated in the anti-leukemic effect of 8-GL.

The role of cAMP in AML signaling and its clinical significance has been previously described. For example, cAMP induces cell growth arrest and enhances chemotherapy-induced AML cell differentiation [66]. In a clinical setting, the administration of theophylline, a stabilizing agent of intracellular cAMP, triggered blast cell clearance and restored normal hematopoiesis in an acute promyelocytic leukemia patient [66]. Despite these advances, the underlying anti-leukemic mechanisms of cAMP signaling are still unclear.

Dysfunctional mTOR signaling is closely involved in AML progression [48]. Here, we confirmed the function of the GPR132-cAMP-PKA-mTOR signaling axis in the regulation of AML cell differentiation. Notably, we also showed that GPR132 agonism by 8GL acts synergistically with an mTOR inhibitor. Given the moderate anti-leukemic activities of mTOR blockers in preclinical AML models and in clinical trials, [59, 67] a combination of GPR132 agonist and mTOR inhibitor appears to be a potential therapeutic strategy for AML. While the detailed molecular mechanism of the synergistic anti-AML effects of GPR132 agonists and mTOR inhibitors remains to be investigated.

In conclusion, our results demonstrated that genetic or pharmacological activation of the orphan receptor GPR132 induces leukemic cell differentiation, providing a potential new therapeutic approach to treat patients with AML. Our results also demonstrated that GPR132 activation by 8GL acts synergistically with an mTOR inhibitor by co-targeting mTOR signaling in vitro and in vivo. The in vivo safety and efficacy of 8GL warrant consideration of novel, optimized GPR132 agonizts for AML differentiation therapy.

## Supplementary information

Supplemental materials

Full length western blots

Reproducibility checklist

## Data Availability

The data which support the conclusions of this article are available from the corresponding author upon reasonable request.
