# Supplementary material for: Activation of orphan receptor GPR132 induces cell differentiation in acute myeloid leukemia
Source: Cell Death Dis. 2022 Nov 27;13(11):1004. doi: 10.1038/s41419-022-05434-z (PMC9701798; doi:10.1038/s41419-022-05434-z)
Supplement: Supplementary file 1 — Supplemental materials [file 41419_2022_5434_MOESM1_ESM.pdf]

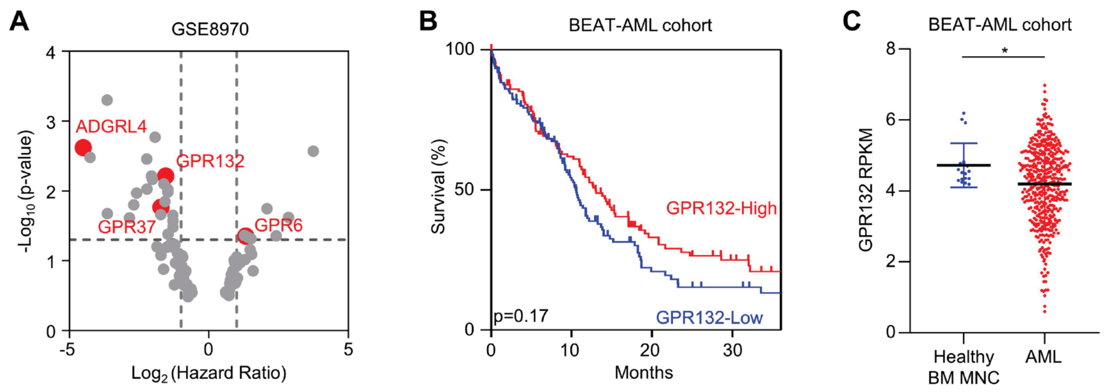


**Supplementary figure 1: related to Figure 1**

(A) Volcano plot depicting hazard ratios (High versus low expressing group) and p-values of orphan GPCRs in AML patients. Data was obtained from GEO database: GSE8970.

(B) Kaplan-Meier plot for AML patients in the BEAT-AML cohort, stratified by median *GPR132* expression (n=138 for High group, n=138 for Low group).

(C) Dot plot depicting *GPR132* expression in AML blasts (n=451) and healthy bone-marrow mononuclear cells (n=19). Data was obtained from the BEAT-AML dataset. Student's t-tests were performed, $*P < 0.05$. BM MNC, bone-marrow mononuclear cell.

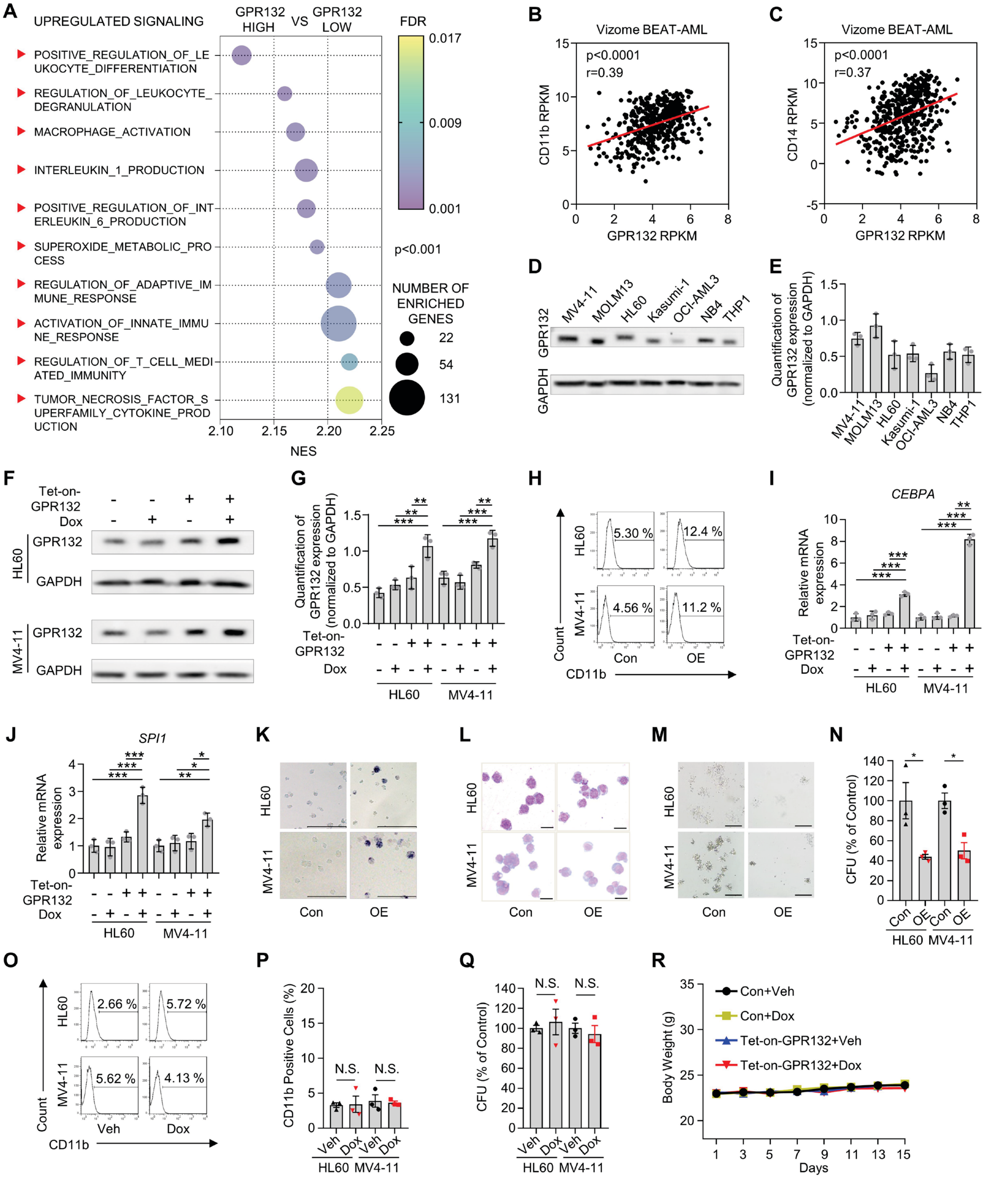


**Supplementary figure 2: related to Figure 2**

(A) Gene Set Enrichment Analysis of TCGA-LAML dataset comparing *GPR132* HIGH and *GPR132* LOW. Scattergrams of gene sets upregulated in *GPR132* HIGH and gene set enrichment plots were shown.

(B-C) Gene expression correlation between *GPR132* and *CD11b* (B) or *CD14* (C) in AML (BEAT-

AML dataset). Two-sided Pearson's correlation analysis was performed.

(D-E) Western blotting (D) and corresponding quantification (E) of GPR132 expression in a panel of AML cell lines. Three independent experiments were performed. Data were shown as mean ± SD (n=3).

(F-G) Western blotting (F) and corresponding quantification (G) of GPR132 expression in Tet-On AML cells. HL60 and MV4-11 cells were treated with or without doxycycline (1 μg/mL) for 48 h. *GPR132* Tet-On AML cells were established by stably transfecting with the Tet-On expression vector for inducible *GPR132* overexpression. Three independent experiments were performed. Data were shown as mean ± SD (n=3). One-way analysis of variance (ANOVA) with Tukey's multiple comparison tests were performed (n = 3), $**P < 0.01$, $***P < 0.001$.

(H) Representative flow cytometric histogram of myeloid differentiation marker CD11b expression using Tet-On AML cells (HL60 and MV4-11) with (OE) or without (Con) doxycycline treatment (1 μg/mL) for 72 h.

(I-J) Boxplots of the mRNA expression of *CEBPA* (I) and *SPI1* (J) using Tet-On AML cells (HL60 and MV4-11) with (OE) or without (Con) doxycycline treatment (1 μg/mL) for 12 h. One-way ANOVA with Tukey's multiple comparison tests were performed, $*P < 0.05$, $**P < 0.01$, $***P < 0.001$.

(K) NBT reduction analysis showing formazan formation in Tet-On AML cells (HL60 and MV4-11) with (OE) or without (Con) doxycycline exposure (1 μg/mL) for 72 h. Scale bars represent 80 μm.

(L) Representative Wright–Giemsa staining images showing the mature myeloid cell morphology of Tet-On AML cells (HL60 and MV4-11) following doxycycline treatment (1 μg/mL, 72 h). Scale bars represent 20 μm.

(M) Representative images of colony formation for Tet-On AML cells. Cells were cultured in semi-solid medium and treated with 1 μg/mL doxycycline to induce GPR132 overexpression. Colonies were photographed under a microscope on day 7. Scale bars represent 400 μm.

(N) Quantification of colony formation assay in Figure 2G. Data shown as mean ± SEM (n=3). Student's t-tests were performed, $*P < 0.05$.

(O) Representative flow cytometric histogram of myeloid differentiation marker CD11b expression using AML cells (HL60 and MV4-11) with (OE) or without (Con) doxycycline treatment (1 μg/mL) for 72 h.

(P) Doxycycline (1 μg/mL, 48 h) do not affect CD11b expression in HL60 and MV4-11 as revealed by Flow-cytometry analysis. Data shown as mean ± SEM (n=3). Student's t-tests were performed, N.S., non-significant.

(Q) Doxycycline (1 μg/mL) do not affect colony formation ability of AML cell lines (HL60 and MV4-11). Data shown as mean ± SEM (n=3). Student's t-tests were performed, N.S., non-significant.

(R) Body weight of mice bearing xenografted tumor during 2-week treatment. Data shown as mean ± SEM (n=6).

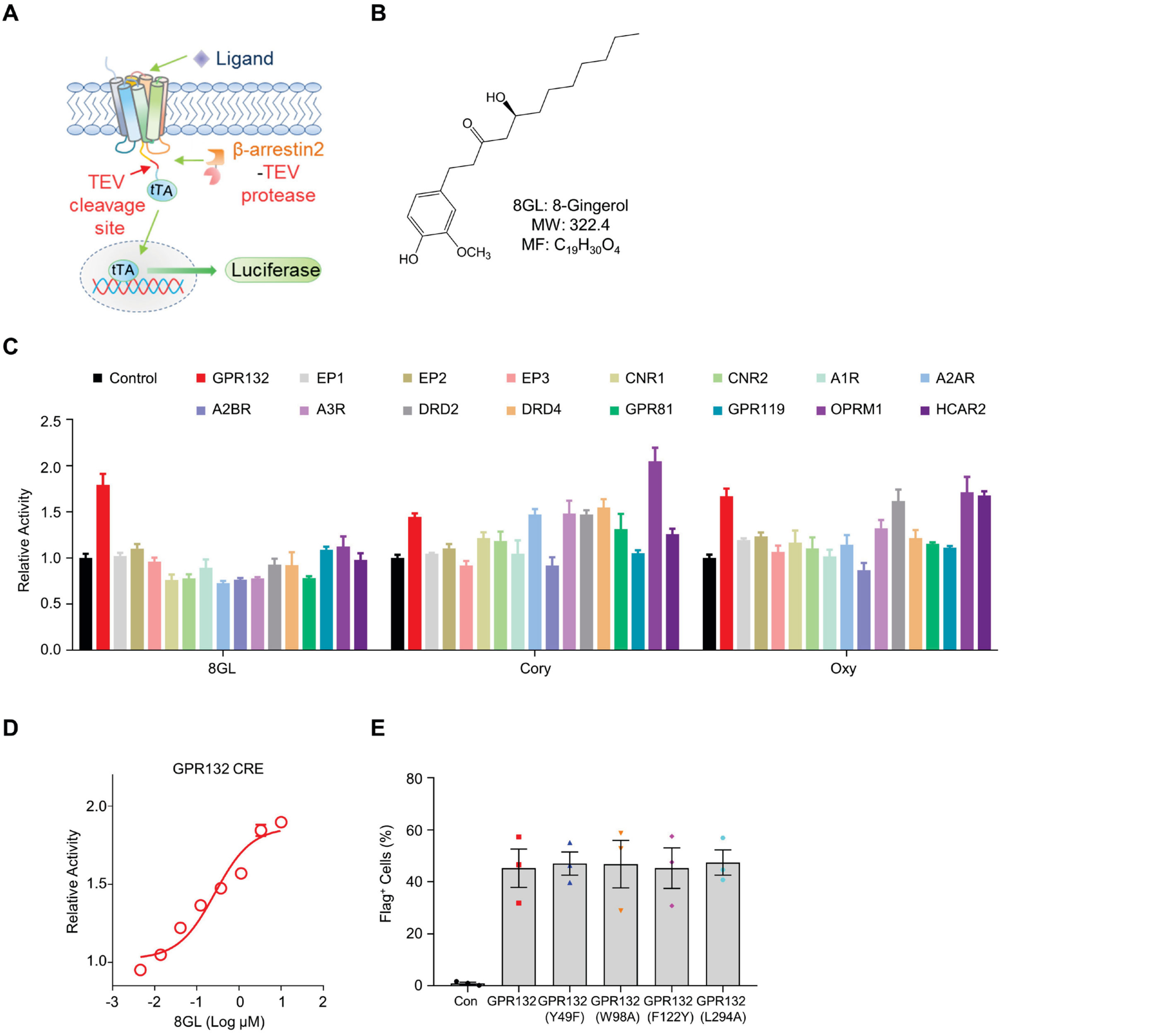


**Supplementary figure 3: related to Figure 3**

(A) Schematic illustration of the Tango assay. Ligand binding to the target receptor stimulates recruitment of the β-arrestin2-TEV protease fusion, triggering release of the tethered transcription factor tTA. The free tTA enters the nucleus and initiates expression of a reporter gene.

(B) Chemical structure of 8-Gingerol (8GL).

(C) Agonistic activity of 8-Gingerol (8GL), oxyresveratrol (Oxy) and corynoline (Cory) for GPCRs in Tango assay. Data was shown as mean ± SEM (n = 3).

(D) Dose-response curve of 8GL for GPR132 activation (EC50 = 0.44 μM) in CRE reporter-gene assays. Data are shown as means ± SEM (n=3).

(E) Flow-cytometry analysis of cell-surface expression of Flag-GPR132 (with or without mutation). CHO cells were transfected with Flag-GPR132 plasmids, and anti-Flag was used to determine cell-surface level of GPR132 after 24 h. Data was shown as mean ± SEM (n = 3).

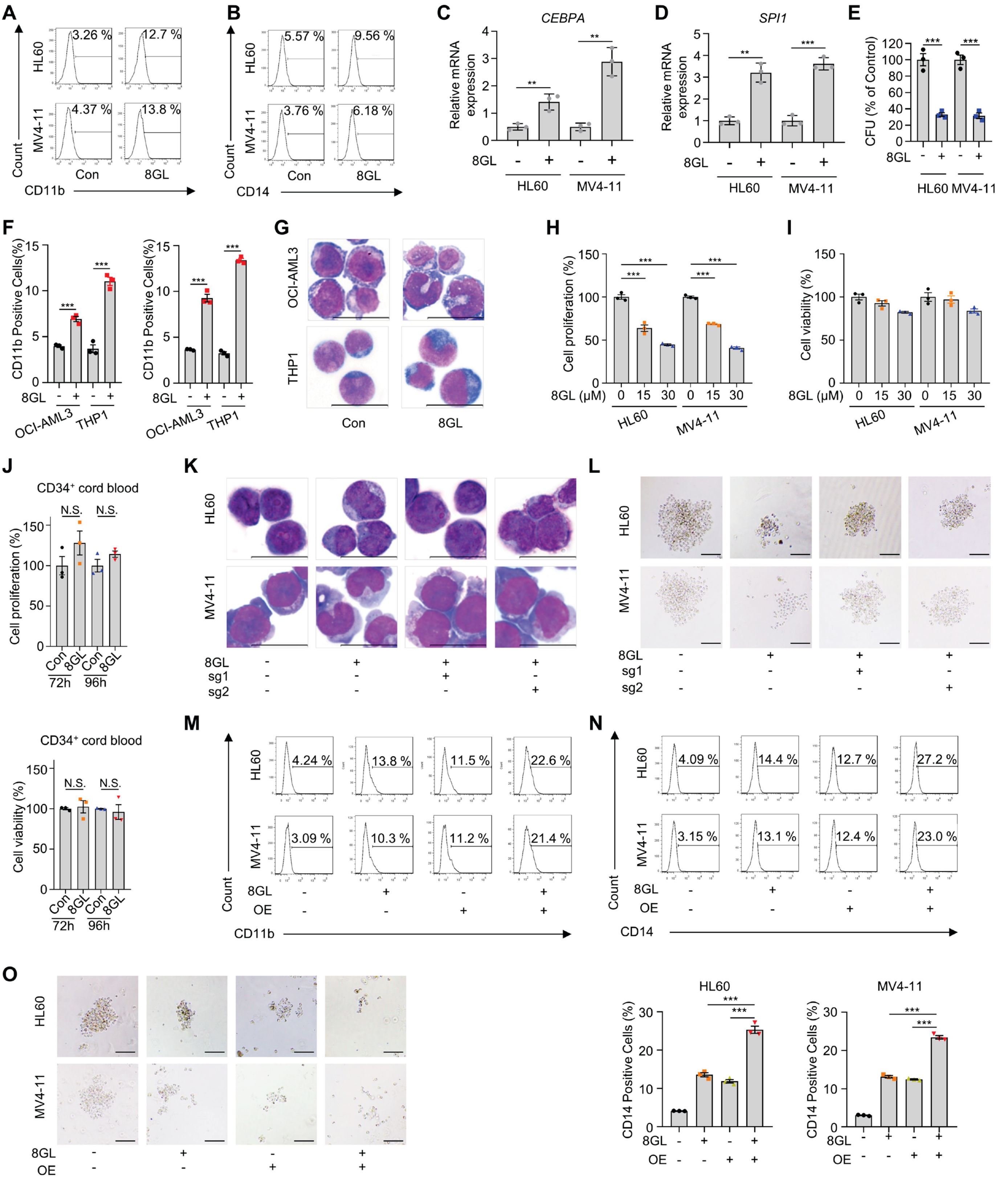

**Supplementary figure 4: related to Figure 4**

(A-B) Representative flow cytometric histogram of the expression of cell surface markers CD11b (A) and CD14 (B) in HL60 and MV4-11 cells 72 h after 8GL treatment (30 μM).

(C-D) Boxplots of the mRNA expression of *CEBPA* (C) and *SPI1* (D) in AML cells (HL60 and MV4-11) treated with or without 30 μM 8GL for 12 h. One-way ANOVA with Tukey's multiple comparison

tests were performed, **P < 0.01, ***P < 0.001.

(E) Quantification of colony formation at Day 9 in Figure 4E. Data was shown as mean ± SEM (n = 3). Unpaired Student's t-tests were performed, ***P < 0.001.

(F-G) OCI-AML3 and THP1 were incubated with 8GL (30 μM) for 72 h and cell differentiation were determined by Flow-cytometry analysis (F) and Wright-Giemsa staining (G). Data are presented as mean ± SEM (n = 3). Unpaired Student's t-tests were performed, ***P < 0.001. Scale bars represent 20 μm.

(H-I) Boxplots of proliferation (H) and viability (I) of HL60 and MV4-11 cells treated with indicated concentrations of 8GL for 72 h (n=3). One-way ANOVA with Tukey's multiple comparison tests were performed, ***P < 0.001.

(J) Cell proliferation (upper panel) and cell viability (lower panel) of $CD34^+$ human cord blood cells treated with 0 μM or 30 μM 8GL for 72 h or 96 h (n=3). Student's t-tests were performed. N.S., non-significant.

(K) Representative Wright-Giemsa staining results showing the mature myeloid cell morphology of *GPR132* wild-type (WT) and *GPR132* knockout (KO) AML cell lines. *GPR132*-WT and *GPR132*-KO cells were treated with 8GL with a concentration of 30 μM for 72 h. Scale bars represent 20 μm. Sg1 and sg2 represent two distinct sgRNAs for *GPR132*.

(L) Representative colony formation images of *GPR132*-WT and *GPR132*-KO AML cell lines treated with or without 8GL. 8GL treatment: 15 μM for 7 days. Colonies were photographed under a microscope on day 7. Scale bars represent 100 μm. Sg1 and sg2 represent two distinct sgRNAs targeting on *GPR132*.

(M) Representative flow cytometric histogram of the expression of cell surface markers CD11b in Tet-On AML cells treated with or without 8GL (30 μM for 72 h) in the presence or absence of doxycycline (1 μg/mL).

(N) Representative flow cytometric histogram (upper panel) and quantification (lower panel) of the expression of cell surface markers CD14 in Tet-On AML cells treated with or without 8GL (30 μM for 72 h) in the presence or absence of doxycycline (1 μg/mL). One-way ANOVA with Tukey's multiple comparison tests were performed (n = 3), ***P < 0.001.

(O) Representative colony formation images of Tet-On AML cells incubated with or without 8GL (15 μM for 7 days) in the presence or absence of doxycycline (1 μg/mL). Colonies were photographed under a microscope on day 7. Scale bars represent 100 μm.

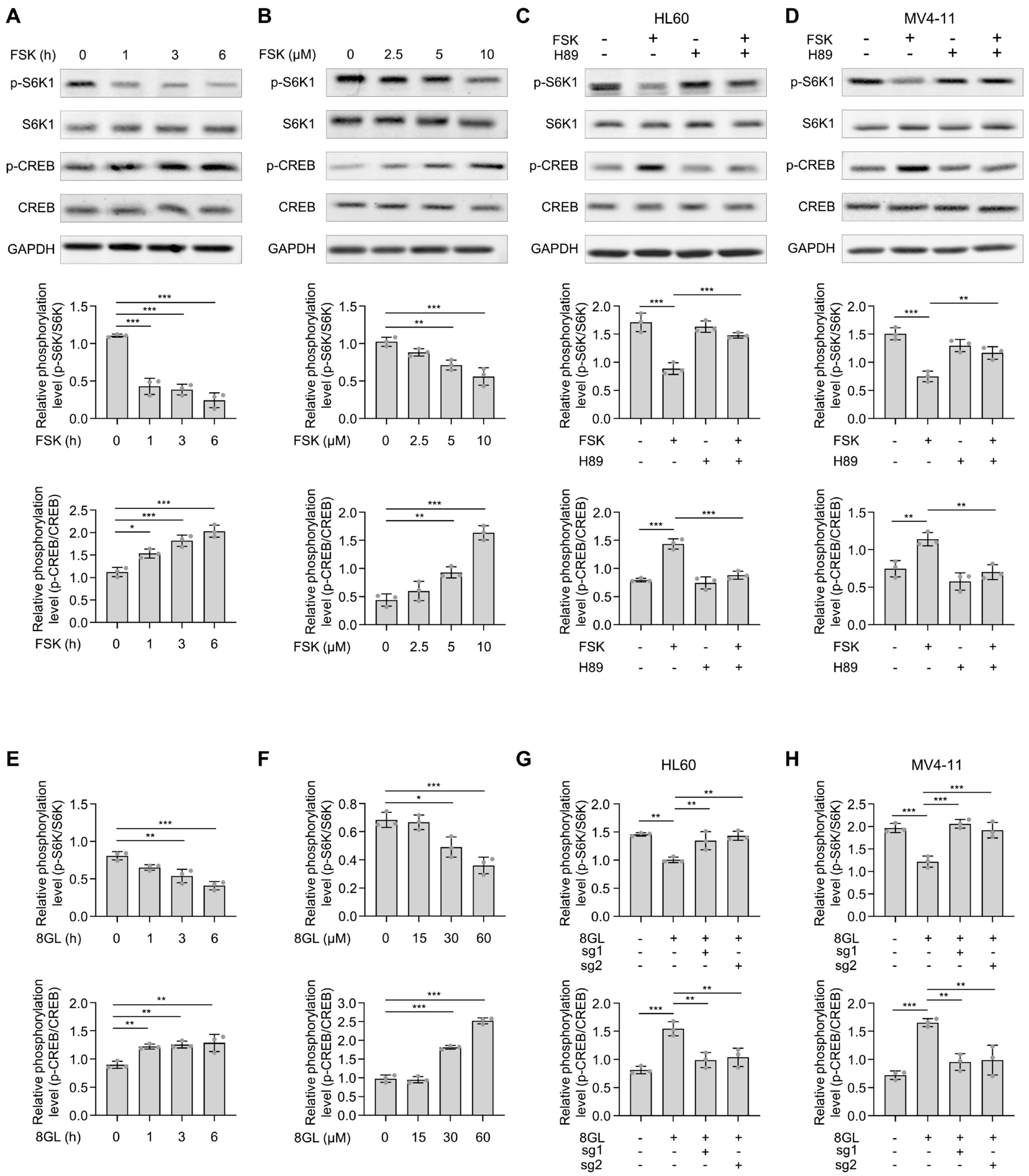


**Supplementary figure 5: related to Figure 5**

(A) Western blotting (upper panel) and corresponding quantification (middle and lower panel) of p-S6K1 (Thr389) and p-CREB (Ser133) in HL60 cells treated with 10 μM FSK. Three independent experiments were performed. Data are shown as mean ± SD (n = 3). One-way ANOVA with Tukey's multiple comparison tests were performed, *P<0.05, ***P < 0.001.

(B) Western blotting (upper panel) and corresponding quantification (middle and lower panel) of p-S6K1 (Thr389) and p-CREB (Ser133) in HL60 cells treated with indicating concentrations of FSK for 3 h. Three independent experiments were performed. Data are shown as mean ± SD (n = 3). One-way ANOVA with Tukey's multiple comparison tests were performed, *P<0.05, **P<0.01, ***P < 0.001.

(C-D) Western blotting (upper panel) and corresponding quantification (middle and lower panel) of p-S6K1 (Thr389) and p-CREB (Ser133) in HL60 cells (C) and MV4-11 cells (D) treated with 10 μM FSK and/or 1 μM FSK for 3 h. Three independent experiments were performed. Data are shown as mean ± SD (n = 3). One-way ANOVA with Tukey's multiple comparison tests were performed, *P<0.05, **P<0.01, ***P < 0.001.

(E) Quantification of western blotting of p-S6K1 (Thr389, upper panel) and p-CREB (Ser133, lower panel) in HL60 cells treated with 8GL (30 μM) for indicated times. Three independent experiments were performed. Data are shown as mean ± SD (n = 3). One-way ANOVA with Tukey's multiple comparison tests were performed, *P<0.05, **P<0.01, ***P < 0.001.

(F) Quantification of western blotting of p-S6K1 (Thr389, upper panel) and p-CREB (Ser133, lower panel) in HL60 cells treated with 8GL for indicated concentrations. Three independent experiments were performed. Data are shown as mean ± SD (n = 3). One-way ANOVA with Tukey's multiple comparison tests were performed, *P<0.05, **P<0.01, ***P < 0.001.

(G-H) Quantification of western blotting of p-S6K1 (Thr389, upper panel) and p-CREB (Ser133, lower panel) in 8GL-treated *GPR132* WT and *GPR132* KO HL60 cells (G) and MV4-11 cells (H). 8GL treatment: 30 μM for 3 h. Sg1 and sg2 represent two distinct sgRNAs targeting on *GPR132*. Three independent experiments were performed. Data are shown as mean ± SD (n = 3). One-way ANOVA with Tukey's multiple comparison tests were performed, *P<0.05, **P<0.01, ***P < 0.001.

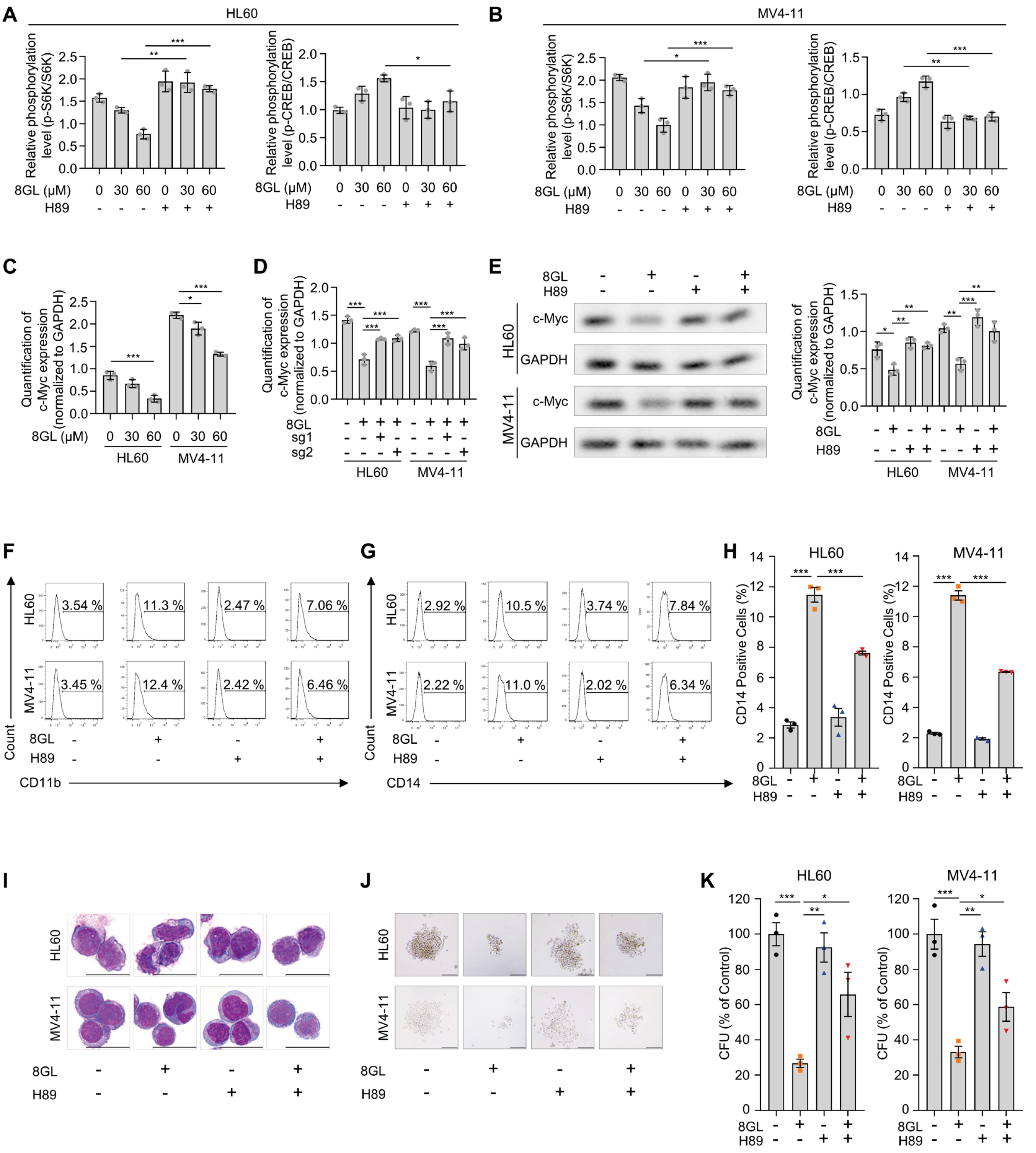


## Supplementary figure 6: related to Figure 5

(A-B) Quantification of western blotting of p-S6K1 (Thr389, upper panel) and p-CREB (Ser133, lower panel) in HL60 cells (A) and MV4-11 cells (B) incubated with 8GL (0 μM, 30 μM or 60 μM) and/or H89 (1 μM). Three independent experiments were performed. Data are shown as mean ± SD (n = 3). One-way ANOVA with Tukey's multiple comparison tests were performed, *P<0.05,

**P<0.01, ***P < 0.001.

(C) Quantification of western blotting of c-Myc in HL60 cells and MV4-11 cells incubated with 8GL (0 μM, 30 μM or 60 μM) for 48 h. Three independent experiments were performed. Data are shown as mean ± SD (n = 3). One-way ANOVA with Tukey's multiple comparison tests were performed, *P<0.05, **P<0.01, ***P < 0.001.

(D) Quantification of western blotting of c-Myc in *GPR132* WT and *GPR132* KO HL60 cells and MV4-11 cells incubated with 8GL (0 μM, 30 μM) for 48 h. Three independent experiments were performed. Sg1 and sg2 represent two distinct sgRNAs targeting on *GPR132*. Data are shown as mean ± SD (n = 3). One-way ANOVA with Tukey's multiple comparison tests were performed, *P<0.05, **P<0.01, ***P < 0.001.

(E) Western blotting (left panel) and corresponding quantification (right panel) of c-Myc expression in 8GL (30 μM) and/or H89 (1 μM) treated HL60 cells and MV4-11 cells (48 h). Three independent experiments were performed. Data are shown as mean ± SD (n = 3). One-way ANOVA with Tukey's multiple comparison tests were performed, *P<0.05, **P<0.01, ***P < 0.001.

(F) Representative flow cytometric histogram of the expression of cell surface markers CD11b in AML cells treated with or without 30 μM 8GL and/or 1 μM H89 for 72 h.

(G-H) Representative flow cytometric histograms (G) and quantification (H) of CD14 expression in AML cells treated with or without 30 μM 8GL and/or 1 μM H89 for 72 h. Data are presented as mean ± SEM (n = 3). One-way ANOVA with Tukey's multiple comparison tests were performed, ***P < 0.001.

(I) Representative Wright-Giemsa staining analysis of AML cells treated with or without 30 μM 8GL and/or 1 μM H89 for 72 h. Scale bars represent 20 μm.

(J-K) Representative colony formation images (J) and quantification (K) of HL60 and MV4-11 cells treated with or without 30 μM 8GL and/or 1 μM H89 for 7 Day. Scale bars represent 100 μm. Right: quantification of colony formation. Data are shown as mean ± SD (n = 3). Unpaired Student’s t-tests were performed, *P < 0.05, **P < 0.01, ***P < 0.001.

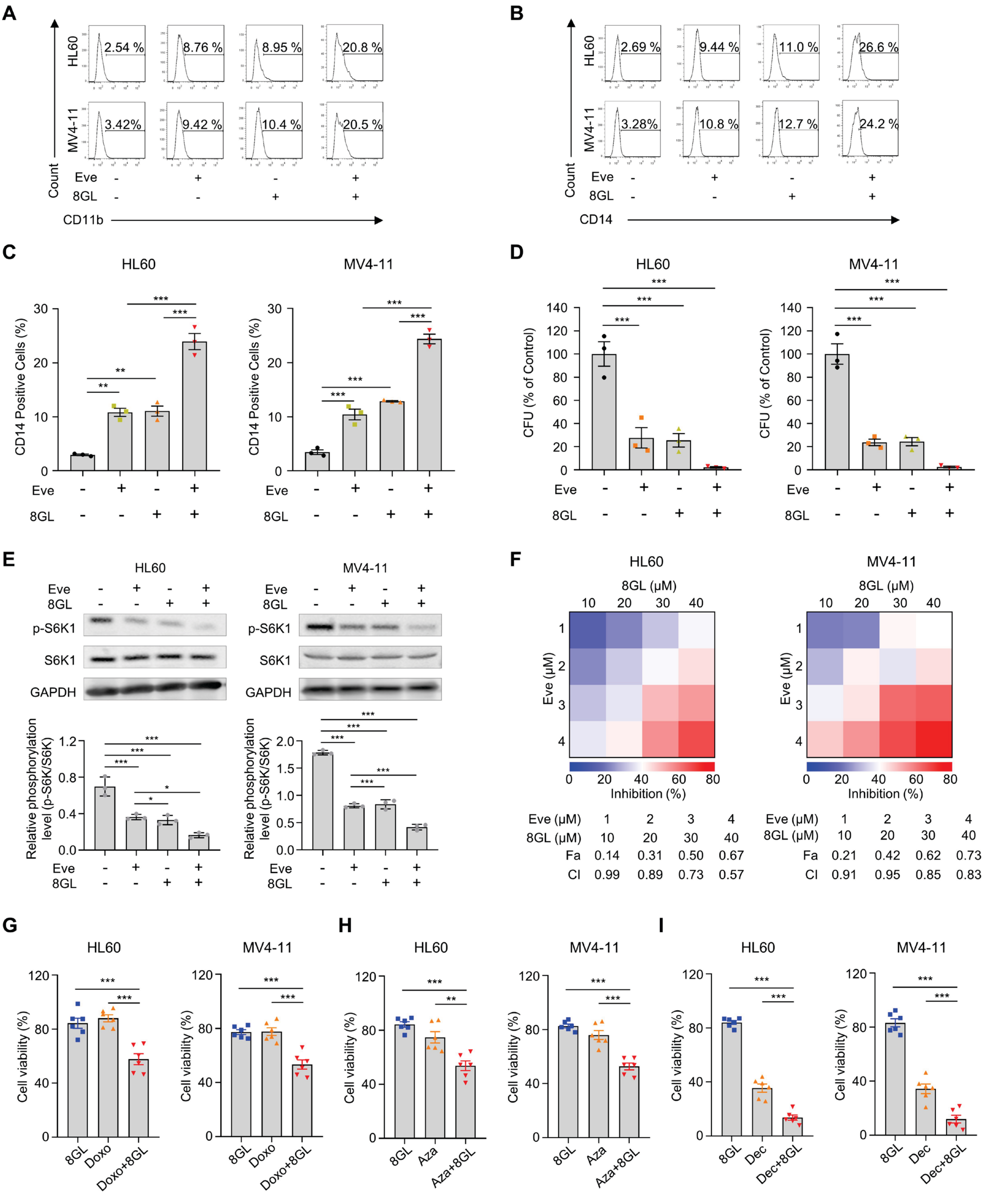


**Supplementary figure 7: related to Figure 6**

(A) Representative flow cytometric histograms of CD11b expression in AML cells treated with vehicle, 30 μM 8GL, 3 μM everolimus (Eve), or drug combination for 72 h.

(B-C) Representative flow cytometric histograms (B) and quantification (C) of CD14 expression in

HL60 and MV4-11 cells treated with vehicle, 8GL (30 μM) and/or Eve (3 μM) for 72 h. Data are presented as mean ± SEM (n = 3). One-way ANOVA with Tukey's multiple comparison tests were performed, ***P < 0.001.

(D) Quantification of colony formation in Figure 6D. Data are shown as mean ± SEM (n = 3). One-way ANOVA with Tukey's multiple comparison tests were performed, ***P < 0.001.

(E) Western blotting (upper panel) and corresponding quantification (lower panel) of p-S6K1 (Thr389) in HL60 cells and MV4-11 cells treated with vehicle, 8GL (30 μM) and/or Eve (3 μM). Three independent experiments were performed. Data are shown as mean ± SD (n = 3). One-way ANOVA with Tukey's multiple comparison tests were performed, *P<0.05, **P<0.01, ***P < 0.001.

(F) Heat map showing inhibition rate of combination between 8GL and Eve. Combination index (CI) were determined by CalcuSyn software (Version 2.1; Biosoft). CI value <1 indicates synergistic. HL60 and MV4-11 cells were treated with the indicated concentrations of 8GL, Eve, or 8GL plus Eve for 72 h and proliferation inhibition was measured using the MTS cell proliferation colorimetric Assay Kit.

(G) HL60 (left panel) and MV4-11 (right panel) cells were incubated with indicated single drug (8GL, 30 μM; doxorubicin, 0.05 μM) or their combination for 72 h, and cell viability was determined by MTS assay. Data are shown as mean ± SEM (n = 6). One-way ANOVA with Tukey's multiple comparison tests were performed, ***P < 0.001. DOXO, doxorubicin.

(H) HL60 (left panel) and MV4-11 (right panel) cells were incubated with indicated single drug (8GL, 30 μM; azacytidine, 1 μM) or their combination for 48 h, and cell viability was determined by MTS assay. Data are shown as mean ± SEM (n = 6). One-way ANOVA with Tukey's multiple comparison tests were performed, ***P < 0.001. Aza, azacytidine.

(I) HL60 (left panel) and MV4-11 (right panel) cells were incubated with indicated single drug (8GL, 30 μM; decitabine, 1 μM) or their combination for 72 h, and cell viability was determined by MTS assay. Data are shown as mean ± SEM (n = 6). One-way ANOVA with Tukey's multiple comparison tests were performed, ***P < 0.001. Dec, decitabine.

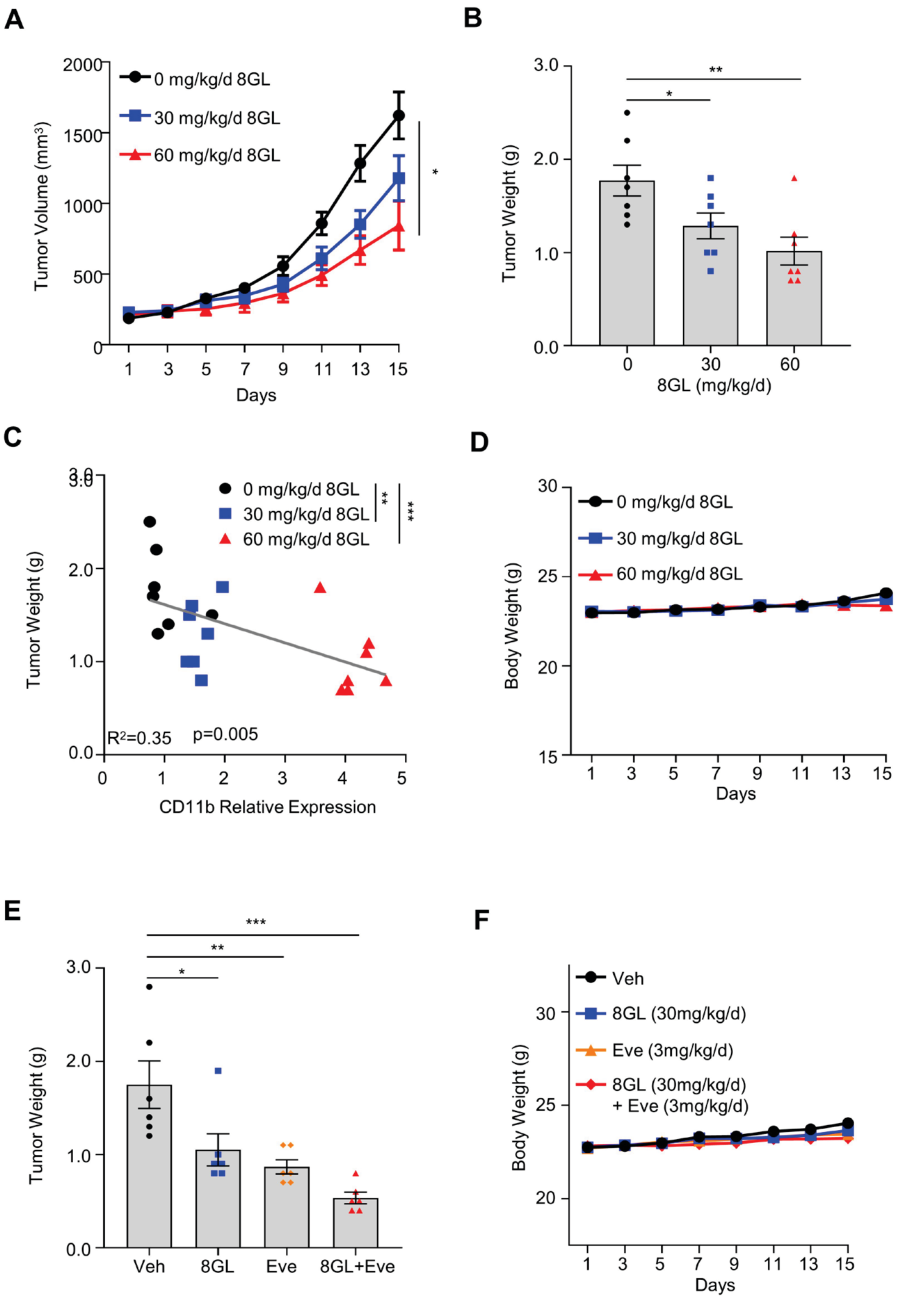


**Supplementary figure 8: related to Figure 6**

(A) 8GL delayed growth and promoted the differentiation of AML cells *in vivo*. HL60 cells were injected subcutaneously into the mid-right flank of female athymic nude mice. When tumor volumes reached almost 200 $mm^3$, the mice were treated with 8GL by intraperitoneal injection for 2 weeks. Data are presented as mean ± SEM (n = 7). One-way ANOVA with Tukey's multiple comparison tests were performed, *P < 0.05.

(B) Mean tumor weight of a subcutaneous HL60 xenograft model on day 15. Data are shown as mean ± SEM (n = 7). Student's t-tests were performed, *P < 0.05, **P < 0.01.

(C) Correlation analysis between tumor weight and *GPR132* expression detected using qPCR. Student's t-tests were performed (n = 6), **P < 0.01, ***P < 0.001.

(D) Body weight of HL60-xenografted mice during time in treatment. Data are shown as mean ± SEM (n = 7).

(E) Tumor weight of a subcutaneous HL60 xenograft model on day 15 treated as in Figure 6E. Data are shown as mean ± SEM (n = 6). One-way ANOVA with Tukey's multiple comparison tests were performed, *P < 0.05, **P < 0.01, ***P < 0.001.

(F) Body weight of HL60-xenografted mice during time in treatment. Data are shown as mean ± SEM (n = 6).

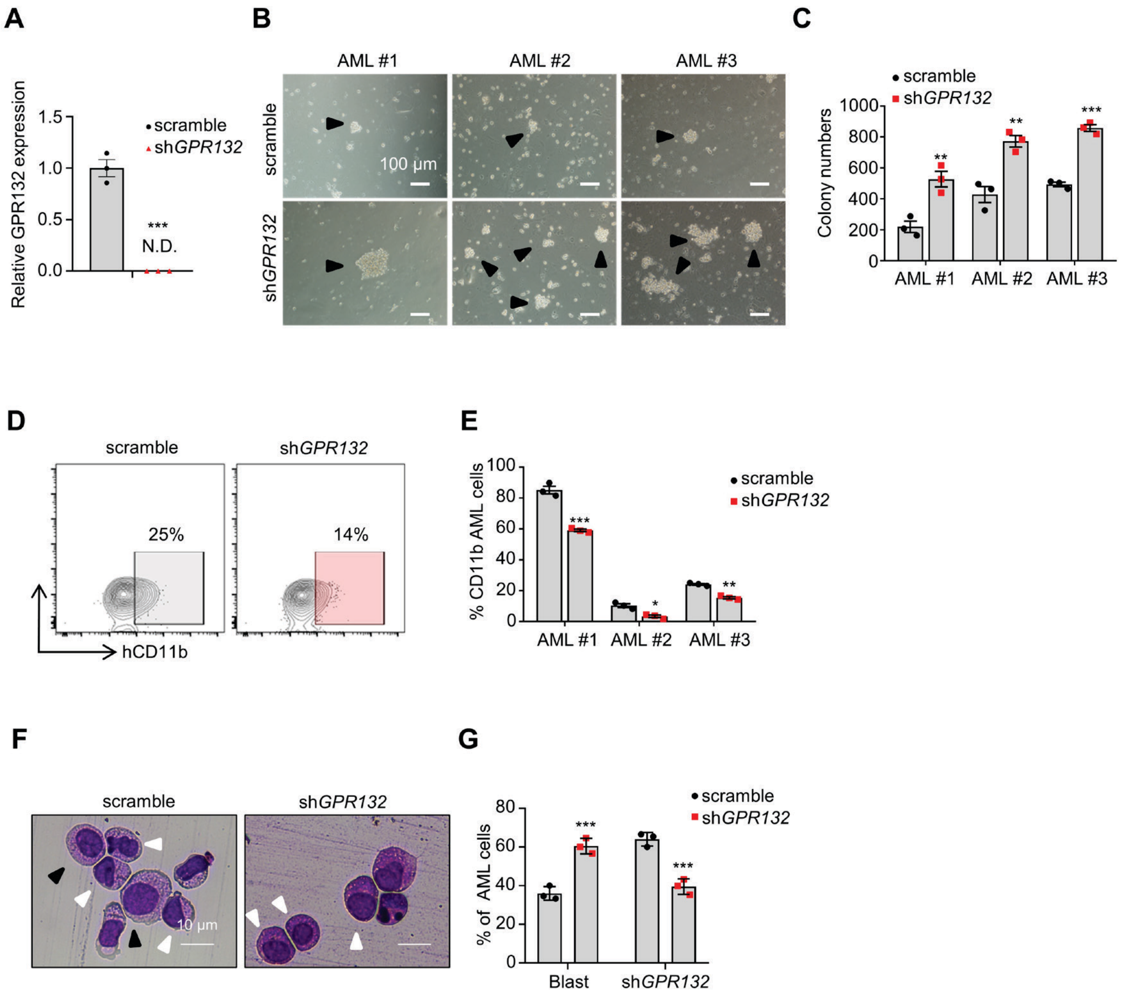


**Supplementary figure 9: related to Figure 7**

(A) Quantification of relative *GPR132* mRNA expression in U937 cells treated with sh*GPR132* or a scrambled control via quantitative RT-PCR. Data are shown as mean ± SEM (n = 3). Student's t-tests were performed, P*** < 0.001.N.D. represents not detected.

(B-C) Representative images (B) and quantitative data (C) for the colony numbers of *GPR132*-knockdown (sh*GPR132*) or control (scrambled) human primary AML cells. Data are shown as mean ± SEM (n = 3). Student's t-tests were performed, P** < 0.01, P*** < 0.001.

(D-E) Representative contour plots (D) and quantitative data (E) by flow cytometric analysis for the percentages of CD11b$^{+}$ cells in *GPR132*-knockdown (sh*GPR132*) or control (scrambled) human primary AML cells. Data are shown as mean ± SEM (n = 3). Student's t-tests were performed, *P<0.05, **P<0.01, ***P<0.001.

(F). Representative images of Wright-Giemsa staining of *GPR132*-knockdown (sh*GPR132*) and control (scrambled) human primary AML cells.

(G) Quantification data of the percentages of blast cells (black arrowhead) and differentiated cells (mature cells, white arrowheads) as shown in panel F. A total of 20-30 cells were counted for each

section and 8-10 sections were evaluated. Data are shown as mean ± SEM ($n = 3$). Student's t-tests were performed, P*** < 0.001.

**Supplementary table 1: The list of orphan GPCRs (except olfactory receptors)**

| Orpha GPCRs | | | | | | | |
|---|---|---|---|---|---|---|---|
| Class A (Rhodopsin-like family) | | | | | Class B (Secretin/adhesion family) | | Class C (Metabotropic family) |
| BRS3 | GPR35 | GPR87 | GPR173 | TAAR2 | ADGRA1 | ADGRF5 | GPR156 |
| GPR3 | GPR37 | GPR88 | GPR174 | TAAR3 | ADGRA2 | ADGRG1 | GPR158 |
| GPR4 | GPR37L1 | GPR101 | GPR176 | TAAR4P | ADGRA3 | ADGRG2 | GPR179 |
| GPR6 | GPR39 | GPR119 | GPR182 | TAAR5 | ADGRB1 | ADGRG3 | GPRC5A |
| GPR42 | GPR45 | GPR132 | GPR183 | TAAR6 | ADGRB2 | ADGRG4 | GPRC5B |
| GPR12 | GPR50 | GPR135 | LGR4 | TAAR8 | ADGRB3 | ADGRG5 | GPRC5C |
| GPR15 | GPR52 | GPR139 | LGR5 | TAAR9 | CELSR1 | ADGRG6 | GPRC5D |
| GPR17 | GPR55 | GPR141 | LGR6 | | CELSR2 | ADGRG7 | GPRC6A |
| GPR18 | GPR61 | GPR142 | MAS1 | | CELSR3 | ADGRL1 | |
| GPR19 | GPR62 | GPR146 | MAS1L | | ADGRD1 | ADGRL2 | |
| GPR20 | GPR63 | GPR148 | MRGPRD | | ADGRD2 | ADGRL3 | |
| GPR21 | GPR65 | GPR149 | MRGPRE | | ADGRE1 | ADGRL4 | |
| GPR22 | GPR68 | GPR150 | MRGPRF | | ADGRE2 | ADGRV1 | |
| GPR25 | GPR75 | GPR151 | MRGPRG | | ADGRE3 | | |
| GPR26 | GPR78 | GPR152 | MRGPRX1 | | ADGRE4P | | |
| GPR27 | GPR79 | GPR153 | MRGPRX2 | | ADGRE5 | | |
| GPR31 | GPR82 | GPR160 | MRGPRX3 | | ADGRF1 | | |
| GPR32 | GPR83 | GPR161 | MRGPRX4 | | ADGRF2 | | |
| GPR33 | GPR84 | GPR162 | P2RY8 | | ADGRF3 | | |
| GPR34 | GPR85 | GPR171 | P2RY10 | | ADGRF4 | | |

**Supplementary table 2: The Hazard Ratio of orphan GPCRs in AML**

| GSE12417-GPL570 | | | | |
|---|---|---|---|---|
| Gene | Hazard Ratio (HR) | P-value | $Log_2HR$ | $-Log_{10}$(P-value) |
| LPHN3 | 3.1480 | 0.0005 | 1.6544 | 3.3010 |
| CD97 | 2.5640 | 0.0006 | 1.3584 | 3.2218 |
| GPR171 | 0.3004 | 0.0007 | -1.7350 | 3.1549 |
| GPR56 | 7.3060 | 0.0007 | 2.8691 | 3.1549 |
| GPR114 | 4.7360 | 0.0016 | 2.2437 | 2.7959 |
| GPCRC6A | 6.7050 | 0.0018 | 2.7452 | 2.7447 |
| GPR146 | 0.3981 | 0.0028 | -1.3288 | 2.5528 |
| P2RY10 | 0.4140 | 0.0030 | -1.2723 | 2.5229 |
| GPR37 | 0.2966 | 0.0035 | -1.7534 | 2.4559 |
| LGR6 | 0.4275 | 0.0041 | -1.2260 | 2.3872 |
| MAS1 | 0.4394 | 0.0054 | -1.1864 | 2.2676 |
| GPR123 | 2.2490 | 0.0065 | 1.1693 | 2.1871 |
| GPR132 | 0.2236 | 0.0088 | -2.1610 | 2.0555 |
| GPR20 | 0.4588 | 0.0102 | -1.1241 | 1.9914 |
| GPR75 | 3.2840 | 0.0102 | 1.7155 | 1.9914 |
| BAI2 | 0.2812 | 0.0157 | -1.8303 | 1.8041 |
| GPR32 | 0.4735 | 0.0167 | -1.0786 | 1.7773 |
| GPR6 | 2.3490 | 0.0194 | 1.2320 | 1.7122 |
| LGR5 | 0.2596 | 0.0228 | -1.9456 | 1.6421 |
| GPR50 | 0.4841 | 0.0236 | -1.0466 | 1.6271 |
| ELTD1 | 0.4864 | 0.0247 | -1.0398 | 1.6073 |
| BRS3 | 2.2100 | 0.0250 | 1.1440 | 1.6021 |
| GPR150 | 0.5083 | 0.0258 | -0.9762 | 1.5884 |
| GPR37L1 | 0.4834 | 0.0258 | -1.0487 | 1.5884 |
| GPR85 | 1.9410 | 0.0258 | 0.9568 | 1.5884 |
| GPR162 | 4.2000 | 0.0263 | 2.0704 | 1.5800 |
| BAI3 | 2.3060 | 0.0266 | 1.2054 | 1.5751 |
| GPR31 | 0.3141 | 0.0292 | -1.6707 | 1.5346 |
| GPRC5D | 0.5154 | 0.0334 | -0.9562 | 1.4763 |
| CELSR1 | 0.5329 | 0.0373 | -0.9081 | 1.4283 |
| GPR15 | 2.0440 | 0.0390 | 1.0314 | 1.4089 |
| GPR173 | 0.5499 | 0.0425 | -0.8628 | 1.3716 |
| LGR4 | 2.2950 | 0.0431 | 1.1985 | 1.3655 |
| LPHN2 | 0.5455 | 0.0461 | -0.8743 | 1.3363 |
| GPR63 | 2.7930 | 0.0469 | 1.4818 | 1.3288 |

| | | | | |
|---|---|---|---|---|
| GPRC5B | 0.4852 | 0.0476 | -1.0433 | 1.3224 |
| GPR68 | 0.2978 | 0.0497 | -1.7476 | 1.3036 |
| EMR2 | 2.5380 | 0.0520 | 1.3437 | 1.2840 |
| GPR97 | 1.7840 | 0.0549 | 0.8351 | 1.2604 |
| GPR112 | 0.4598 | 0.0554 | -1.1209 | 1.2565 |
| GPR160 | 1.7810 | 0.0565 | 0.8327 | 1.2480 |
| TAAR5 | 0.5106 | 0.0576 | -0.9697 | 1.2396 |
| GPR83 | 1.9870 | 0.0579 | 0.9906 | 1.2373 |
| GPR35 | 0.5173 | 0.0637 | -0.9509 | 1.1959 |
| GPR25 | 0.4825 | 0.0665 | -1.0514 | 1.1772 |
| GPR17 | 0.3696 | 0.0682 | -1.4360 | 1.1662 |
| GPR19 | 2.0560 | 0.0712 | 1.0398 | 1.1475 |
| GPR26 | 0.5109 | 0.0750 | -0.9689 | 1.1249 |
| GPR125 | 2.5360 | 0.0839 | 1.3426 | 1.0762 |
| GPR126 | 0.6035 | 0.0891 | -0.7286 | 1.0501 |
| GPR52 | 0.4993 | 0.0902 | -1.0020 | 1.0448 |
| GPR110 | 0.5282 | 0.0924 | -0.9208 | 1.0343 |
| GPR18 | 0.3719 | 0.0927 | -1.4270 | 1.0329 |
| GPR113 | 1.8990 | 0.0931 | 0.9252 | 1.0311 |
| GPR45 | 0.5487 | 0.0992 | -0.8659 | 1.0035 |
| GPR135 | 1.6940 | 0.1003 | 0.7604 | 0.9987 |
| GPR174 | 1.6850 | 0.1041 | 0.7527 | 0.9825 |
| GPR182 | 0.6240 | 0.1075 | -0.6804 | 0.9686 |
| GPR21 | 1.6840 | 0.1101 | 0.7519 | 0.9582 |
| EMR1 | 0.5394 | 0.1114 | -0.8906 | 0.9531 |
| GPR128 | 2.1630 | 0.1125 | 1.1130 | 0.9488 |
| GPRC5C | 1.9340 | 0.1138 | 0.9516 | 0.9439 |
| GPRC5A | 1.6180 | 0.1145 | 0.6942 | 0.9412 |
| GPR27 | 1.6100 | 0.1159 | 0.6871 | 0.9359 |
| GPR161 | 0.6262 | 0.1193 | -0.6753 | 0.9234 |
| GPR55 | 1.8260 | 0.1226 | 0.8687 | 0.9115 |
| GPR61 | 0.5985 | 0.1227 | -0.7406 | 0.9112 |
| GPR98 | 2.0330 | 0.1233 | 1.0236 | 0.9090 |
| GPR156 | 0.6120 | 0.1385 | -0.7084 | 0.8586 |
| BAI1 | 2.1080 | 0.1407 | 1.0759 | 0.8517 |
| GPR22 | 0.6221 | 0.1424 | -0.6848 | 0.8465 |
| GPR153 | 0.4494 | 0.1549 | -1.1539 | 0.8099 |
| GPR111 | 0.6644 | 0.1625 | -0.5899 | 0.7891 |
| GPR3 | 1.6520 | 0.1724 | 0.7242 | 0.7635 |

| | | | | |
|---|---|---|---|---|
| GPR124 | 1.5350 | 0.1791 | 0.6182 | 0.7469 |
| GPR133 | 0.6764 | 0.1892 | -0.5641 | 0.7231 |
| GPR176 | 0.5806 | 0.1903 | -0.7844 | 0.7206 |
| GPR39 | 1.5350 | 0.1907 | 0.6182 | 0.7196 |
| GPR64 | 1.4630 | 0.1943 | 0.5489 | 0.7115 |
| GPR84 | 1.5670 | 0.1994 | 0.6480 | 0.7003 |
| GPR42 | 1.5880 | 0.2053 | 0.6672 | 0.6876 |
| GPR116 | 1.6610 | 0.2172 | 0.7321 | 0.6631 |
| TAAR2 | 1.4640 | 0.2234 | 0.5499 | 0.6509 |
| GPR183 | 0.5949 | 0.2532 | -0.7493 | 0.5965 |
| GPR4 | 0.7000 | 0.2533 | -0.5146 | 0.5964 |
| GPR34 | 0.6235 | 0.2609 | -0.6815 | 0.5835 |
| GPR115 | 1.7160 | 0.2670 | 0.7790 | 0.5735 |
| CELSR2 | 0.6566 | 0.2696 | -0.6069 | 0.5693 |
| GPR87 | 1.3760 | 0.2791 | 0.4605 | 0.5542 |
| MAS1L | 1.3750 | 0.2990 | 0.4594 | 0.5243 |
| GPR88 | 0.7454 | 0.3779 | -0.4239 | 0.4226 |
| GPR65 | 1.5720 | 0.4150 | 0.6526 | 0.3820 |
| GPR12 | 1.4710 | 0.4397 | 0.5568 | 0.3568 |
| LPHN1 | 1.2520 | 0.4695 | 0.3242 | 0.3284 |
| CELSR3 | 1.2670 | 0.4799 | 0.3414 | 0.3188 |
| EMR3 | 0.7533 | 0.5055 | -0.4087 | 0.2963 |
| **GSE12417-GPL96** | | | | |
| **Gene** | **Hazard Ratio (HR)** | **P-value** | **Log$_2$HR** | **- Log$_{10}$(P-value)** |
| GPR114 | 2.0630 | 0.0011 | 1.0447 | 2.9586 |
| GPR65 | 0.4899 | 0.0011 | -1.0294 | 2.9586 |
| GPR19 | 0.5276 | 0.0018 | -0.9225 | 2.7447 |
| GPR132 | 0.5524 | 0.0031 | -0.8562 | 2.5086 |
| TAAR5 | 2.4580 | 0.0032 | 1.2975 | 2.4949 |
| GPR174 | 2.6580 | 0.0033 | 1.4103 | 2.4815 |
| GPR12 | 1.7700 | 0.0041 | 0.8237 | 2.3872 |
| GPR115 | 1.9500 | 0.0043 | 0.9635 | 2.3665 |
| LPHN2 | 2.9210 | 0.0047 | 1.5465 | 2.3279 |
| GPR150 | 1.8930 | 0.0056 | 0.9207 | 2.2518 |
| GPR75 | 2.0500 | 0.0111 | 1.0356 | 1.9547 |
| GPR156 | 1.7360 | 0.0114 | 0.7958 | 1.9431 |
| GPR6 | 1.6710 | 0.0135 | 0.7407 | 1.8697 |
| GPR56 | 1.6980 | 0.0140 | 0.7638 | 1.8539 |

| | | | | |
|---|---|---|---|---|
| GPR35 | 0.6075 | 0.0146 | -0.7190 | 1.8356 |
| GPR37 | 0.5550 | 0.0148 | -0.8494 | 1.8297 |
| GPR26 | 0.6024 | 0.0149 | -0.7312 | 1.8268 |
| GPR18 | 0.6157 | 0.0153 | -0.6997 | 1.8153 |
| GPR173 | 1.7680 | 0.0161 | 0.8221 | 1.7932 |
| GPR113 | 1.8050 | 0.0174 | 0.8520 | 1.7595 |
| GPR124 | 0.5413 | 0.0186 | -0.8855 | 1.7305 |
| GPR110 | 0.6033 | 0.0201 | -0.7291 | 1.6968 |
| GPR27 | 0.5770 | 0.0247 | -0.7934 | 1.6073 |
| GPR116 | 1.9120 | 0.0270 | 0.9351 | 1.5686 |
| CELSR1 | 0.6484 | 0.0289 | -0.6250 | 1.5391 |
| GPR63 | 0.6437 | 0.0316 | -0.6355 | 1.5003 |
| GPR3 | 1.5240 | 0.0342 | 0.6079 | 1.4660 |
| GPR183 | 0.6561 | 0.0350 | -0.6080 | 1.4559 |
| GPR52 | 1.6210 | 0.0433 | 0.6969 | 1.3635 |
| ELTD1 | 0.6120 | 0.0460 | -0.7084 | 1.3372 |
| BAI1 | 0.5378 | 0.0528 | -0.8949 | 1.2774 |
| EMR3 | 0.6614 | 0.0529 | -0.5964 | 1.2765 |
| GPR87 | 1.5200 | 0.0538 | 0.6041 | 1.2692 |
| GPRC5D | 0.6669 | 0.0664 | -0.5845 | 1.1778 |
| GPR34 | 0.5944 | 0.0684 | -0.7505 | 1.1649 |
| GPR31 | 0.5944 | 0.0685 | -0.7505 | 1.1643 |
| CD97 | 1.4730 | 0.0712 | 0.5588 | 1.1475 |
| LPHN1 | 1.4280 | 0.0737 | 0.5140 | 1.1325 |
| GPR153 | 1.7390 | 0.0789 | 0.7983 | 1.1029 |
| LGR4 | 0.6945 | 0.0875 | -0.5260 | 1.0580 |
| GPRC5C | 1.4220 | 0.0886 | 0.5079 | 1.0526 |
| EMR1 | 1.5660 | 0.0887 | 0.6471 | 1.0521 |
| BAI2 | 0.6110 | 0.0888 | -0.7108 | 1.0516 |
| GPR50 | 0.6650 | 0.0914 | -0.5886 | 1.0391 |
| GPR135 | 1.4080 | 0.0915 | 0.4936 | 1.0386 |
| GPR146 | 0.6506 | 0.0950 | -0.6202 | 1.0223 |
| GPR160 | 0.6848 | 0.0968 | -0.5462 | 1.0141 |
| GPR55 | 1.3920 | 0.1019 | 0.4772 | 0.9918 |
| GPR161 | 1.4820 | 0.1047 | 0.5675 | 0.9801 |
| GPR45 | 1.3900 | 0.1056 | 0.4751 | 0.9763 |
| GPR98 | 1.3850 | 0.1078 | 0.4699 | 0.9674 |
| P2RY10 | 0.7219 | 0.1081 | -0.4701 | 0.9662 |
| GPR20 | 0.7308 | 0.1145 | -0.4525 | 0.9412 |

| | | | | |
|---|---|---|---|---|
| GPR176 | 1.3920 | 0.1172 | 0.4772 | 0.9311 |
| GPR25 | 1.7630 | 0.1176 | 0.8180 | 0.9296 |
| GPR162 | 1.5270 | 0.1258 | 0.6107 | 0.9003 |
| GPR171 | 1.5140 | 0.1329 | 0.5984 | 0.8765 |
| GPR123 | 0.6260 | 0.1389 | -0.6758 | 0.8573 |
| LGR5 | 1.5440 | 0.1474 | 0.6267 | 0.8315 |
| GPR37L1 | 0.7250 | 0.1497 | -0.4639 | 0.8248 |
| LPHN3 | 0.6993 | 0.1641 | -0.5160 | 0.7849 |
| GPR21 | 1.6260 | 0.1649 | 0.7013 | 0.7828 |
| GPR61 | 1.3980 | 0.1668 | 0.4834 | 0.7778 |
| GPR32 | 1.4840 | 0.1683 | 0.5695 | 0.7739 |
| LGR6 | 1.4040 | 0.1848 | 0.4895 | 0.7333 |
| GPR4 | 0.7499 | 0.1997 | -0.4152 | 0.6996 |
| GPR17 | 0.7026 | 0.2023 | -0.5092 | 0.6940 |
| GPR68 | 1.3100 | 0.2095 | 0.3896 | 0.6788 |
| GPR15 | 1.3190 | 0.2100 | 0.3994 | 0.6778 |
| GPR85 | 0.7799 | 0.2277 | -0.3586 | 0.6426 |
| BAI3 | 1.3070 | 0.2521 | 0.3863 | 0.5984 |
| MAS1 | 1.4080 | 0.2522 | 0.4936 | 0.5983 |
| EMR2 | 1.4500 | 0.2550 | 0.5361 | 0.5935 |
| GPR64 | 0.7906 | 0.2551 | -0.3390 | 0.5933 |
| GPR83 | 0.8080 | 0.2850 | -0.3076 | 0.5452 |
| GPRC5A | 1.3750 | 0.2923 | 0.4594 | 0.5342 |
| GPR133 | 0.7752 | 0.2937 | -0.3674 | 0.5321 |
| GPR88 | 0.7421 | 0.3079 | -0.4303 | 0.5116 |
| GPR22 | 1.2200 | 0.3183 | 0.2869 | 0.4972 |
| GPRC5B | 0.7331 | 0.3309 | -0.4479 | 0.4803 |
| MAS1L | 1.2600 | 0.3313 | 0.3334 | 0.4798 |
| GPR84 | 0.8279 | 0.3385 | -0.2725 | 0.4704 |
| GPR42 | 1.3080 | 0.3666 | 0.3874 | 0.4358 |
| GPR39 | 0.8367 | 0.3935 | -0.2572 | 0.4051 |
| TAAR2 | 1.1850 | 0.4089 | 0.2449 | 0.3884 |
| CELSR3 | 0.8110 | 0.4615 | -0.3022 | 0.3358 |
| GPR126 | 0.8230 | 0.4711 | -0.2810 | 0.3269 |
| GPR182 | 0.8298 | 0.5135 | -0.2692 | 0.2895 |

| | | | | |
|---|---|---|---|---|
| CELSR2 | 0.8600 | 0.5486 | -0.2176 | 0.2607 |
| **GSE8970** | | | | |
| **Gene** | **Hazard Ratio (HR)** | **P-value** | **$Log_2HR$** | **$-Log_{10}$(P-value)** |
| GPR25 | 0.0799 | 0.0005 | -3.6455 | 3.3010 |
| P2RY10 | 0.2624 | 0.0017 | -1.9302 | 2.7696 |
| ELTD1 | 0.0440 | 0.0024 | -4.5070 | 2.6198 |
| LPHN1 | 0.2137 | 0.0035 | -2.2263 | 2.4559 |
| GPR132 | 0.3420 | 0.0061 | -1.5479 | 2.2147 |
| GPR63 | 0.2405 | 0.0061 | -2.0559 | 2.2147 |
| GPR85 | 0.2465 | 0.0068 | -2.0203 | 2.1675 |
| GPR31 | 0.3257 | 0.0079 | -1.6184 | 2.1024 |
| GPR87 | 0.2144 | 0.0094 | -2.2216 | 2.0269 |
| MAS1 | 0.3593 | 0.0104 | -1.4767 | 1.9830 |
| GPR88 | 0.3364 | 0.0142 | -1.5718 | 1.8477 |
| GPR37 | 0.3039 | 0.0171 | -1.7183 | 1.7670 |
| GPR161 | 0.3015 | 0.0218 | -1.7298 | 1.6615 |
| GPR68 | 0.4076 | 0.0224 | -1.2948 | 1.6498 |
| GPR20 | 0.1392 | 0.0243 | -2.8448 | 1.6144 |
| GPR17 | 0.4094 | 0.0270 | -1.2884 | 1.5686 |
| GPR12 | 0.4086 | 0.0330 | -1.2912 | 1.4815 |
| GPR110 | 0.3621 | 0.0417 | -1.4655 | 1.3799 |
| EMR1 | 2.4160 | 0.0433 | 1.2726 | 1.3635 |
| EMR3 | 5.3120 | 0.0441 | 2.4093 | 1.3556 |
| GPR6 | 2.4920 | 0.0442 | 1.3173 | 1.3546 |
| EMR2 | 2.8480 | 0.0485 | 1.5099 | 1.3143 |
| GPR171 | 0.4289 | 0.0588 | -1.2213 | 1.2306 |
| GPR27 | 0.3816 | 0.0589 | -1.3899 | 1.2299 |
| GPR19 | 0.2750 | 0.0628 | -1.8625 | 1.2020 |
| GPR65 | 0.3516 | 0.0640 | -1.5080 | 1.1938 |
| GPR153 | 0.4190 | 0.0650 | -1.2550 | 1.1871 |
| GPR18 | 2.7970 | 0.0696 | 1.4839 | 1.1574 |
| BRS3 | 0.4573 | 0.0778 | -1.1288 | 1.1090 |
| GPR64 | 2.8900 | 0.0810 | 1.5311 | 1.0915 |
| TAAR2 | 0.5100 | 0.0838 | -0.9714 | 1.0768 |

| | | | | |
|---|---|---|---|---|
| GPR162 | 0.4862 | 0.0864 | -1.0404 | 1.0635 |
| GPRC5C | 0.5210 | 0.0888 | -0.9406 | 1.0516 |
| GPR4 | 2.4590 | 0.0962 | 1.2981 | 1.0168 |
| GPR173 | 1.8760 | 0.0983 | 0.9077 | 1.0074 |
| GPR3 | 2.1850 | 0.1062 | 1.1276 | 0.9739 |
| GPR176 | 1.9090 | 0.1066 | 0.9328 | 0.9722 |
| GPR35 | 0.4379 | 0.1072 | -1.1913 | 0.9698 |
| GPR182 | 0.4584 | 0.1111 | -1.1253 | 0.9543 |
| CD97 | 1.9840 | 0.1151 | 0.9884 | 0.9389 |
| GPR52 | 2.0210 | 0.1176 | 1.0151 | 0.9296 |
| GPR126 | 0.3237 | 0.1324 | -1.6273 | 0.8781 |
| BAI2 | 1.8220 | 0.1388 | 0.8655 | 0.8576 |
| GPR124 | 3.0030 | 0.1392 | 1.5864 | 0.8564 |
| GPR22 | 0.5620 | 0.1401 | -0.8314 | 0.8536 |
| LGR4 | 0.5556 | 0.1454 | -0.8479 | 0.8374 |
| GPR50 | 0.5321 | 0.1456 | -0.9102 | 0.8368 |
| BAI1 | 1.7450 | 0.1583 | 0.8032 | 0.8005 |
| GPR98 | 0.5022 | 0.1636 | -0.9937 | 0.7862 |
| GPR32 | 0.4797 | 0.1646 | -1.0598 | 0.7836 |
| GPR135 | 0.5116 | 0.1678 | -0.9669 | 0.7752 |
| BAI3 | 2.0320 | 0.1764 | 1.0229 | 0.7535 |
| GPR42 | 0.5346 | 0.1787 | -0.9035 | 0.7479 |
| GPRC5B | 1.7250 | 0.1842 | 0.7866 | 0.7347 |
| GPR183 | 1.7110 | 0.1873 | 0.7748 | 0.7275 |
| GPR75 | 1.9330 | 0.2101 | 0.9508 | 0.6776 |
| GPRC5D | 1.6770 | 0.2128 | 0.7459 | 0.6720 |
| LPHN2 | 0.5789 | 0.2171 | -0.7886 | 0.6633 |
| GPRC5A | 0.5797 | 0.2180 | -0.7866 | 0.6615 |
| GPR45 | 0.4252 | 0.2219 | -1.2338 | 0.6538 |
| CELSR1 | 0.5351 | 0.2232 | -0.9021 | 0.6513 |
| GPR21 | 0.5586 | 0.2301 | -0.8401 | 0.6381 |
| TAAR5 | 1.7200 | 0.2351 | 0.7824 | 0.6287 |
| GPR15 | 0.6425 | 0.2545 | -0.6382 | 0.5943 |
| GPR56 | 1.6700 | 0.2614 | 0.7398 | 0.5827 |
| CELSR3 | 0.6514 | 0.2787 | -0.6184 | 0.5549 |

| | | | | |
|---|---|---|---|---|
| GPR116 | 1.5200 | 0.2787 | 0.6041 | 0.5549 |
| LGR5 | 1.5270 | 0.2900 | 0.6107 | 0.5376 |
| GPR39 | 0.6045 | 0.2902 | -0.7262 | 0.5373 |
| GPR37L1 | 1.6490 | 0.3143 | 0.7216 | 0.5027 |
| CELSR2 | 0.5957 | 0.3256 | -0.7473 | 0.4873 |

**Supplementary table 3. Patient information in GSE12417**

| Sample ID | age | Characteristics | OS days | Live status | GPR132 expression |
|---|---|---|---|---|---|
| GSM311695 CN_AML | 40 | FAB M1 | 33 | 1 | 9.1 |
| GSM311716 CN_AML | 67 | FAB M4 | 114 | 0 | 8.8874 |
| GSM311734 CN_AML | 36 | FAB M4 | 326 | 1 | 8.8597 |
| GSM311601 CN_AML | 66 | FAB M4 | 280 | 0 | 8.8278 |
| GSM311669 CN_AML | 62 | FAB M2 | 123 | 1 | 8.8151 |
| GSM311668 CN_AML | 80 | FAB M4 | 27 | 1 | 8.7729 |
| GSM311719 CN_AML | 71 | FAB M4 | 794 | 0 | 8.7548 |
| GSM311713 CN_AML | 75 | FAB M1 | 13 | 1 | 8.7463 |
| GSM311747 CN_AML | 71 | FAB M4 | 1176 | 0 | 8.744 |
| GSM311720 CN_AML | 83 | FAB M2 | 127 | 1 | 8.7051 |
| GSM311683 CN_AML | 72 | FAB M5 | 1176 | 0 | 8.705 |
| GSM311752 CN_AML | 59 | FAB M1 | 1176 | 0 | 8.641 |
| GSM311723 CN_AML | 66 | FAB M2 | 693 | 1 | 8.6353 |
| GSM311757 CN_AML | 56 | FAB M5 | 676 | 0 | 8.627 |
| GSM311646 CN_AML | 19 | FAB M2 | 826 | 0 | 8.6189 |
| GSM311714 CN_AML | 39 | FAB M2 | 51 | 0 | 8.6068 |
| GSM311633 CN_AML | 62 | FAB M2 | 842 | 0 | 8.6041 |
| GSM311681 CN_AML | 39 | FAB M5 | 1176 | 0 | 8.5968 |
| GSM311632 CN_AML | 45 | FAB M4 | 1176 | 0 | 8.5953 |
| GSM311617 CN_AML | 66 | FAB M2 | 8 | 1 | 8.5912 |
| GSM311598 CN_AML | 62 | FAB M4 | 4 | 1 | 8.5531 |
| GSM311729 CN_AML | 20 | FAB M2 | 1176 | 0 | 8.5512 |
| GSM311750 CN_AML | 43 | FAB M2 | 1176 | 0 | 8.5501 |
| GSM311715 CN_AML | 39 | FAB M1 | 608 | 0 | 8.5465 |
| GSM311697 CN_AML | 44 | FAB M4 | 251 | 1 | 8.5364 |
| GSM311694 CN_AML | 75 | FAB M1 | 91 | 1 | 8.5321 |
| GSM311620 CN_AML | 64 | FAB M1 | 223 | 1 | 8.5317 |
| GSM311736 CN_AML | 54 | FAB M4 | 1176 | 0 | 8.527 |
| GSM311688 CN_AML | 47 | FAB M1 | 416 | 1 | 8.5262 |
| GSM311732 CN_AML | 24 | FAB M5 | 79 | 1 | 8.5243 |
| GSM311629 CN_AML | 63 | FAB M1 | 18 | 1 | 8.5192 |
| GSM311710 CN_AML | 57 | FAB M2 | 891 | 0 | 8.5172 |
| GSM311659 CN_AML | 48 | FAB M4 | 31 | 1 | 8.5114 |
| GSM311742 CN_AML | 68 | FAB M1 | 1113 | 0 | 8.5045 |
| GSM311711 CN_AML | 58 | FAB M4 | 850 | 0 | 8.5011 |
| GSM311628 CN_AML | 78 | FAB M2 | 4 | 1 | 8.4982 |
| GSM311748 CN_AML | 40 | FAB M5 | 1176 | 0 | 8.4963 |
| GSM311741 CN_AML | 69 | FAB M2 | 192 | 1 | 8.4958 |
| GSM311627 CN_AML | 54 | FAB M1 | 75 | 1 | 8.4948 |
| GSM311603 CN_AML | 52 | FAB M2 | 657 | 1 | 8.4945 |
| GSM311602 CN_AML | 61 | MDS RAEB | 275 | 0 | 8.4936 |
| GSM311693 CN_AML | 78 | FAB M1 | 62 | 1 | 8.4886 |
| GSM311615 CN_AML | 26 | FAB M1 | 317 | 0 | 8.487 |
| GSM311728 CN_AML | 65 | FAB M4 | 1176 | 0 | 8.4867 |
| GSM311745 CN_AML | 67 | FAB M1 | 1176 | 0 | 8.4861 |
| GSM311634 CN_AML | 28 | FAB M1 | 425 | 0 | 8.4845 |
| GSM311726 CN_AML | 17 | FAB M5 | 1176 | 0 | 8.484 |
| GSM311619 CN_AML | 61 | FAB M1 | 860 | 0 | 8.4838 |
| GSM311666 CN_AML | 71 | FAB M1 | 236 | 1 | 8.4836 |
| GSM311604 CN_AML | 66 | FAB M1 | 427 | 1 | 8.4806 |

| | | | | | |
|---|---|---|---|---|---|
| GSM311709 CN_AML | 61 | FAB M2 | 184 | 1 | 8.4736 |
| GSM311712 CN_AML | 57 | FAB M4 | 575 | 0 | 8.4734 |
| GSM311685 CN_AML | 49 | FAB M1 | 107 | 1 | 8.4629 |
| GSM311687 CN_AML | 40 | FAB M5 | 250 | 1 | 8.4624 |
| GSM311701 CN_AML | 55 | FAB M1 | 229 | 1 | 8.4556 |
| GSM311640 CN_AML | 33 | FAB M2 | 293 | 1 | 8.4555 |
| GSM311651 CN_AML | 72 | FAB M2 | 289 | 1 | 8.4533 |
| GSM311649 CN_AML | 59 | FAB M4 | 1176 | 0 | 8.4511 |
| GSM311631 CN_AML | 41 | FAB M4 | 1176 | 0 | 8.4506 |
| GSM311708 CN_AML | 38 | FAB M1 | 581 | 0 | 8.4475 |
| GSM311680 CN_AML | 67 | FAB M1 | 157 | 1 | 8.4441 |
| GSM311754 CN_AML | 61 | FAB M4 | 1176 | 0 | 8.4383 |
| GSM311718 CN_AML | 49 | FAB M4 | 840 | 0 | 8.4359 |
| GSM311727 CN_AML | 74 | FAB M4 | 284 | 1 | 8.4337 |
| GSM311677 CN_AML | 75 | FAB M1 | 50 | 1 | 8.4329 |
| GSM311755 CN_AML | 61 | FAB M2 | 223 | 1 | 8.4227 |
| GSM311600 CN_AML | 66 | FAB M2 | 432 | 1 | 8.4216 |
| GSM311618 CN_AML | 66 | FAB M4 | 72 | 0 | 8.4192 |
| GSM311698 CN_AML | 53 | FAB M4 | 314 | 1 | 8.4151 |
| GSM311689 CN_AML | 71 | FAB M2 | 289 | 0 | 8.4144 |
| GSM311758 CN_AML | 53 | FAB M4 | 1176 | 0 | 8.4143 |
| GSM311630 CN_AML | 55 | FAB M6 | 1103 | 0 | 8.4139 |
| GSM311621 CN_AML | 62 | FAB M4 | 214 | 1 | 8.4136 |
| GSM311756 CN_AML | 53 | FAB M4 | 51 | 1 | 8.4131 |
| GSM311642 CN_AML | 46 | FAB M4 | 105 | 1 | 8.4129 |
| GSM311654 CN_AML | 62 | FAB M2 | 30 | 1 | 8.4104 |
| GSM311700 CN_AML | 44 | FAB M0 | 741 | 1 | 8.4066 |
| GSM311667 CN_AML | 49 | FAB M2 | 280 | 1 | 8.4042 |
| GSM311730 CN_AML | 70 | FAB M1 | 19 | 1 | 8.403 |
| GSM311724 CN_AML | 59 | FAB M1 | 31 | 1 | 8.4014 |
| GSM311613 CN_AML | 49 | FAB M5 | 209 | 0 | 8.4006 |
| GSM311722 CN_AML | 44 | FAB M4 | 486 | 0 | 8.3922 |
| GSM311637 CN_AML | 47 | FAB M2 | 366 | 0 | 8.3896 |
| GSM311643 CN_AML | 43 | FAB M2 | 119 | 1 | 8.3833 |
| GSM311641 CN_AML | 34 | FAB M0 | 1176 | 0 | 8.3779 |
| GSM311707 CN_AML | 77 | FAB M2 | 18 | 1 | 8.3776 |
| GSM311725 CN_AML | 65 | FAB M2 | 148 | 1 | 8.3736 |
| GSM311733 CN_AML | 67 | FAB M5 | 137 | 1 | 8.3735 |
| GSM311699 CN_AML | 72 | FAB M1 | 176 | 1 | 8.3718 |
| GSM311670 CN_AML | 27 | FAB M4 | 321 | 1 | 8.3689 |
| GSM311661 CN_AML | 46 | FAB M2 | 1176 | 0 | 8.3614 |
| GSM311662 CN_AML | 81 | FAB M1 | 41 | 1 | 8.3562 |
| GSM311655 CN_AML | 43 | FAB M4 | 1176 | 0 | 8.3562 |
| GSM311696 CN_AML | 53 | FAB M1 | 872 | 1 | 8.3525 |
| GSM311622 CN_AML | 61 | FAB M1 | 33 | 1 | 8.3518 |
| GSM311743 CN_AML | 68 | FAB M5 | 559 | 1 | 8.351 |
| GSM311676 CN_AML | 67 | FAB M1 | 39 | 1 | 8.3487 |
| GSM311652 CN_AML | 66 | FAB M5 | 113 | 1 | 8.3482 |
| GSM311626 CN_AML | 74 | FAB M1 | 240 | 1 | 8.3477 |
| GSM311658 CN_AML | 26 | FAB M2 | 44 | 1 | 8.346 |
| GSM311660 CN_AML | 69 | FAB M2 | 86 | 1 | 8.345 |
| GSM311691 CN_AML | 20 | FAB M4 | 340 | 1 | 8.3425 |
| GSM311650 CN_AML | 49 | FAB M4 | 61 | 1 | 8.3369 |

| | | | | | |
|---|---|---|---|---|---|
| GSM311616 CN_AML | 25 | FAB M2 | 190 | 1 | 8.3338 |
| GSM311746 CN_AML | 65 | FAB M1 | 233 | 1 | 8.3327 |
| GSM311749 CN_AML | 38 | FAB M1 | 1176 | 0 | 8.3261 |
| GSM311648 CN_AML | 52 | FAB M2 | 283 | 1 | 8.3259 |
| GSM311706 CN_AML | 69 | FAB M2 | 484 | 1 | 8.3244 |
| GSM311692 CN_AML | 60 | FAB M2 | 452 | 0 | 8.3243 |
| GSM311738 CN_AML | 55 | FAB M2 | 1176 | 0 | 8.3241 |
| GSM311760 CN_AML | 32 | FAB M1 | 916 | 0 | 8.3189 |
| GSM311703 CN_AML | 56 | FAB M1 | 4 | 1 | 8.3172 |
| GSM311653 CN_AML | 54 | FAB M2 | 109 | 1 | 8.3149 |
| GSM311739 CN_AML | 75 | FAB M4 | 41 | 1 | 8.3148 |
| GSM311721 CN_AML | 58 | FAB M4 | 24 | 1 | 8.3136 |
| GSM311610 CN_AML | 64 | FAB M0 | 761 | 1 | 8.3112 |
| GSM311635 CN_AML | 62 | FAB M0 | 257 | 1 | 8.3111 |
| GSM311664 CN_AML | 76 | FAB M2 | 1102 | 1 | 8.3104 |
| GSM311645 CN_AML | 73 | FAB M2 | 260 | 1 | 8.3039 |
| GSM311682 CN_AML | 60 | FAB M1 | 599 | 1 | 8.301 |
| GSM311665 CN_AML | 47 | FAB M6 | 749 | 1 | 8.2867 |
| GSM311656 CN_AML | 40 | FAB M2 | 205 | 1 | 8.2831 |
| GSM311672 CN_AML | 69 | FAB M1 | 124 | 1 | 8.2829 |
| GSM311657 CN_AML | 70 | FAB M1 | 252 | 1 | 8.281 |
| GSM311684 CN_AML | 45 | FAB M1 | 238 | 1 | 8.277 |
| GSM311636 CN_AML | 73 | FAB M4 | 1176 | 0 | 8.2759 |
| GSM311690 CN_AML | 77 | FAB M1 | 28 | 1 | 8.271 |
| GSM311606 CN_AML | 48 | FAB M1 | 41 | 1 | 8.2698 |
| GSM311740 CN_AML | 44 | FAB M2 | 352 | 1 | 8.259 |
| GSM311717 CN_AML | 32 | FAB M2 | 849 | 0 | 8.2557 |
| GSM311644 CN_AML | 65 | FAB M5 | 47 | 1 | 8.2436 |
| GSM311759 CN_AML | 79 | FAB M2 | 175 | 1 | 8.2423 |
| GSM311679 CN_AML | 58 | FAB M4 | 77 | 1 | 8.2296 |
| GSM311638 CN_AML | 34 | FAB M6 | 1176 | 0 | 8.2264 |
| GSM311624 CN_AML | 76 | FAB M6 | 924 | 0 | 8.2261 |
| GSM311753 CN_AML | 29 | FAB M1 | 1176 | 0 | 8.2229 |
| GSM311673 CN_AML | 60 | FAB M2 | 256 | 1 | 8.2176 |
| GSM311671 CN_AML | 52 | FAB M0 | 56 | 1 | 8.2128 |
| GSM311678 CN_AML | 46 | FAB M1 | 17 | 1 | 8.2126 |
| GSM311611 CN_AML | 54 | FAB M5 | 294 | 1 | 8.2126 |
| GSM311647 CN_AML | 63 | FAB M5 | 461 | 1 | 8.2009 |
| GSM311751 CN_AML | 57 | FAB M5 | 1176 | 0 | 8.2008 |
| GSM311744 CN_AML | 62 | FAB M2 | 209 | 0 | 8.1971 |
| GSM311686 CN_AML | 53 | FAB M6 | 175 | 1 | 8.1908 |
| GSM311737 CN_AML | 37 | FAB M4 | 999 | 1 | 8.1897 |
| GSM311704 CN_AML | 47 | FAB M4 | 1176 | 0 | 8.1869 |
| GSM311674 CN_AML | 54 | FAB M4 | 442 | 1 | 8.1821 |
| GSM311599 CN_AML | 59 | FAB M4 | 104 | 0 | 8.179 |
| GSM311675 CN_AML | 71 | FAB M6 | 41 | 1 | 8.1782 |
| GSM311735 CN_AML | 66 | FAB M2 | 304 | 1 | 8.1745 |
| GSM311609 CN_AML | 52 | FAB M4 | 253 | 1 | 8.1734 |
| GSM311625 CN_AML | 76 | FAB M2 | 1176 | 0 | 8.1688 |
| GSM311612 CN_AML | 48 | FAB M5 | 719 | 0 | 8.1516 |
| GSM311663 CN_AML | 47 | FAB M1 | 285 | 0 | 8.1445 |
| GSM311705 CN_AML | 38 | FAB M4 | 271 | 1 | 8.1405 |
| GSM311639 CN_AML | 50 | FAB M2 | 247 | 1 | 8.1385 |

| | | | | | |
|---|---|---|---|---|---|
| GSM311614 CN_AML | 48 | FAB M5 | 190 | 1 | 8.1346 |
| GSM311731 CN_AML | 66 | FAB M4 | 1176 | 0 | 8.1295 |
| GSM311702 CN_AML | 68 | FAB M1 | 263 | 1 | 8.1015 |
| GSM311608 CN_AML | 55 | FAB M4 | 51 | 1 | 8.0699 |
| GSM311605 CN_AML | 34 | FAB M5 | 421 | 1 | 8.0665 |
| GSM311623 CN_AML | 72 | FAB M1 | 3 | 1 | 8.02 |
| GSM311607 CN_AML | 56 | FAB M5 | 1 | 1 | 7.9676 |

**Supplementary table 4. List of primer sequences for quantitative qRT-PCR and shRNAs**

| qRT-PCR primers | Sequences (5' - 3') |
|---|---|
| Human *GPR132*-F | TGCTGCATCTCCTGCGATAG |
| Human *GPR132*-R | TCAAAATAGCTGTCCGGCGT |
| Human *CD11b*-F | GCCTTGACCTTATGTCATGGG |
| Human *CD11b*-R | CCTGTGCTGTAGTCGCACT |
| Human *CEBPA*-F | AGACGTCCATCGACATCAGC |
| Human *CEBPA*-R | AGGAACTCGTCGTTGAAGGC |
| Human *SPI1*-F | GCGACCATTACTGGGACTTCC |
| Human *SPI2*-R | GGGTATCGAGGACGTGCAT |
| shRNAs for *GPR132* | Target sequences (5' - 3') |
| Scrambled | CCTAAGGTTAAGTCGCCCTCG |
| Human sh*GPR132* | CTGGGTCATCTATATCCGCAA |

**Supplementary table 5 Infomations for AML patient samples**

| Sample | Gender | Age | Subtype | Survival | Cytogentics | Mutations |
|---|---|---|---|---|---|---|
| AML#1 | Male | 26 | M5 | 6 months | 46, XY | WT1 |
| AML#2 | Female | 57 | M2 | Alive | 46，X,X | None |
| AML#3 | Male | 49 | M2 | 2 weeks | 46，X,Y | None |
