# Supplementary material for: Activation of orphan receptor GPR132 induces cell differentiation in acute myeloid leukemia
Source: Cell Death Dis. 2022 Nov 27;13(11):1004. doi: 10.1038/s41419-022-05434-z (PMC9701798; doi:10.1038/s41419-022-05434-z)

# Figure 4F

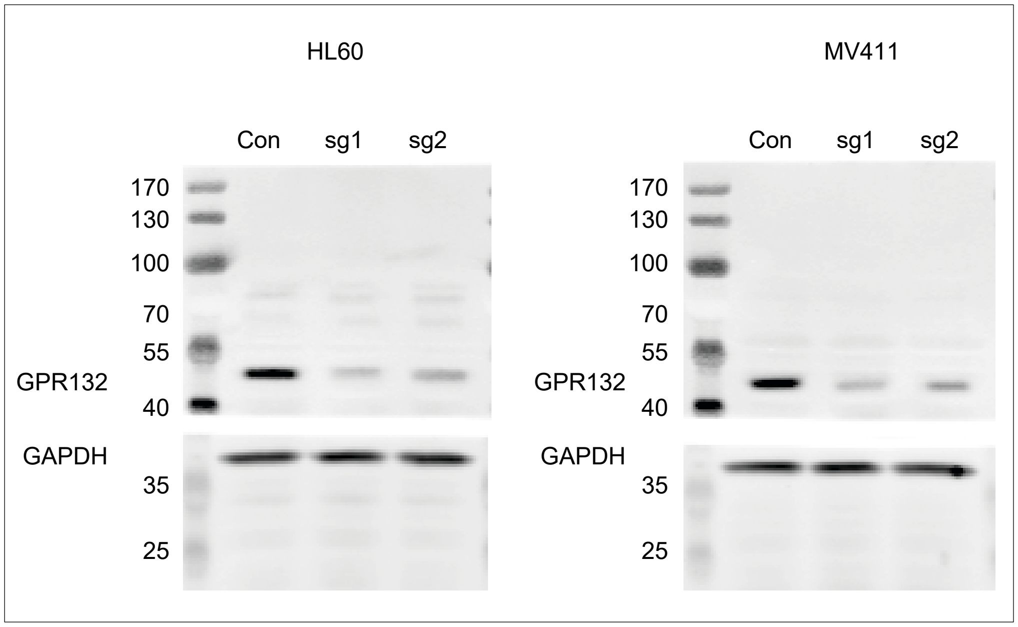
HL60
Con
sg1
sg2
170
130
100
70
55
GPR132
40
GAPDH
35
25
MV411
Con
sg1
sg2
170
130
100
70
55
GPR132
40
GAPDH
35
25

## Figure 5B

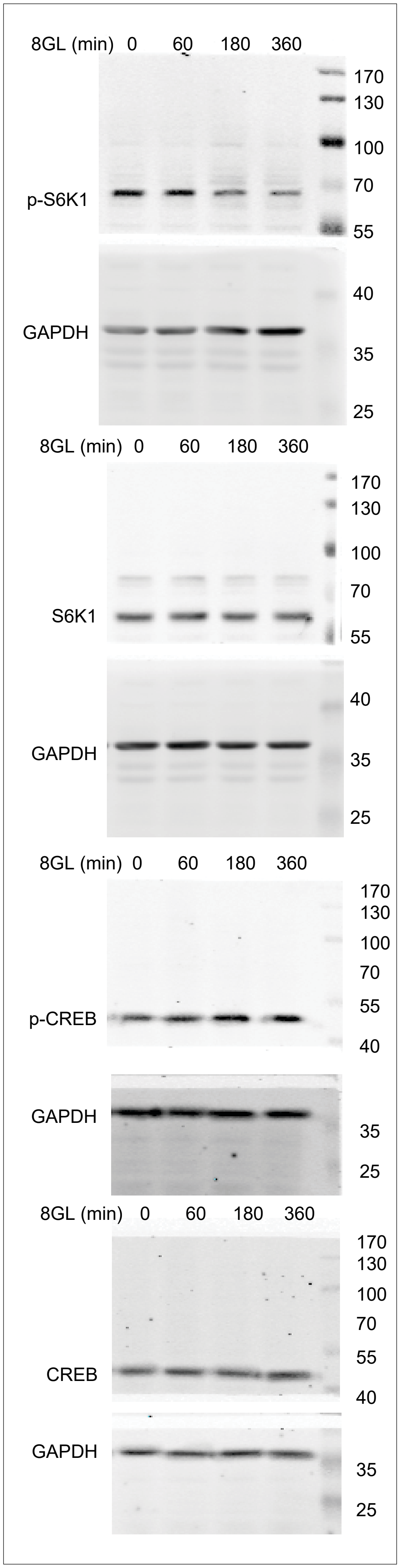


## Figure 5C

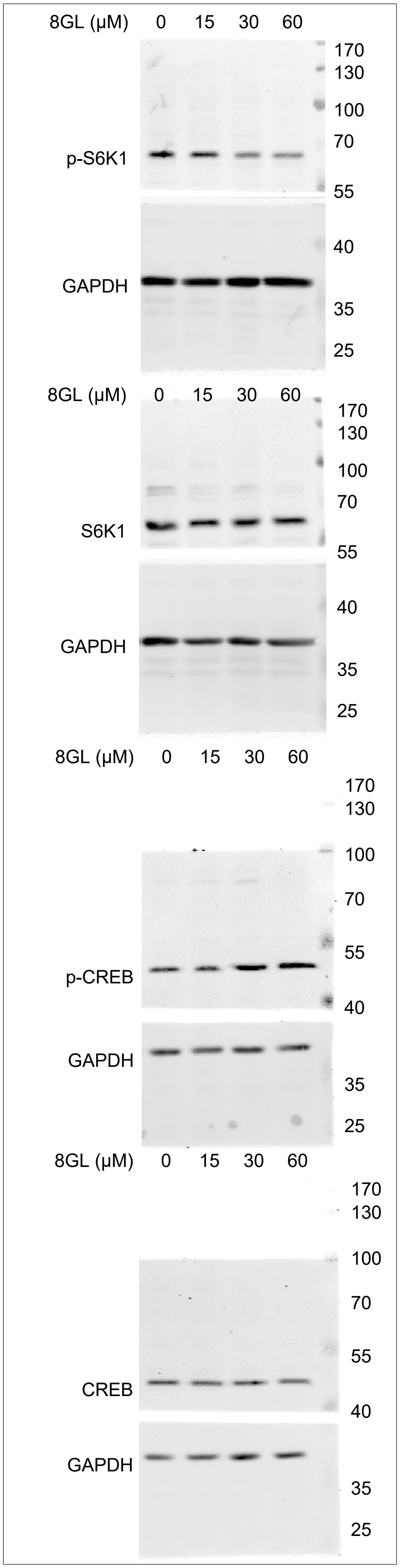

# Figure 5D

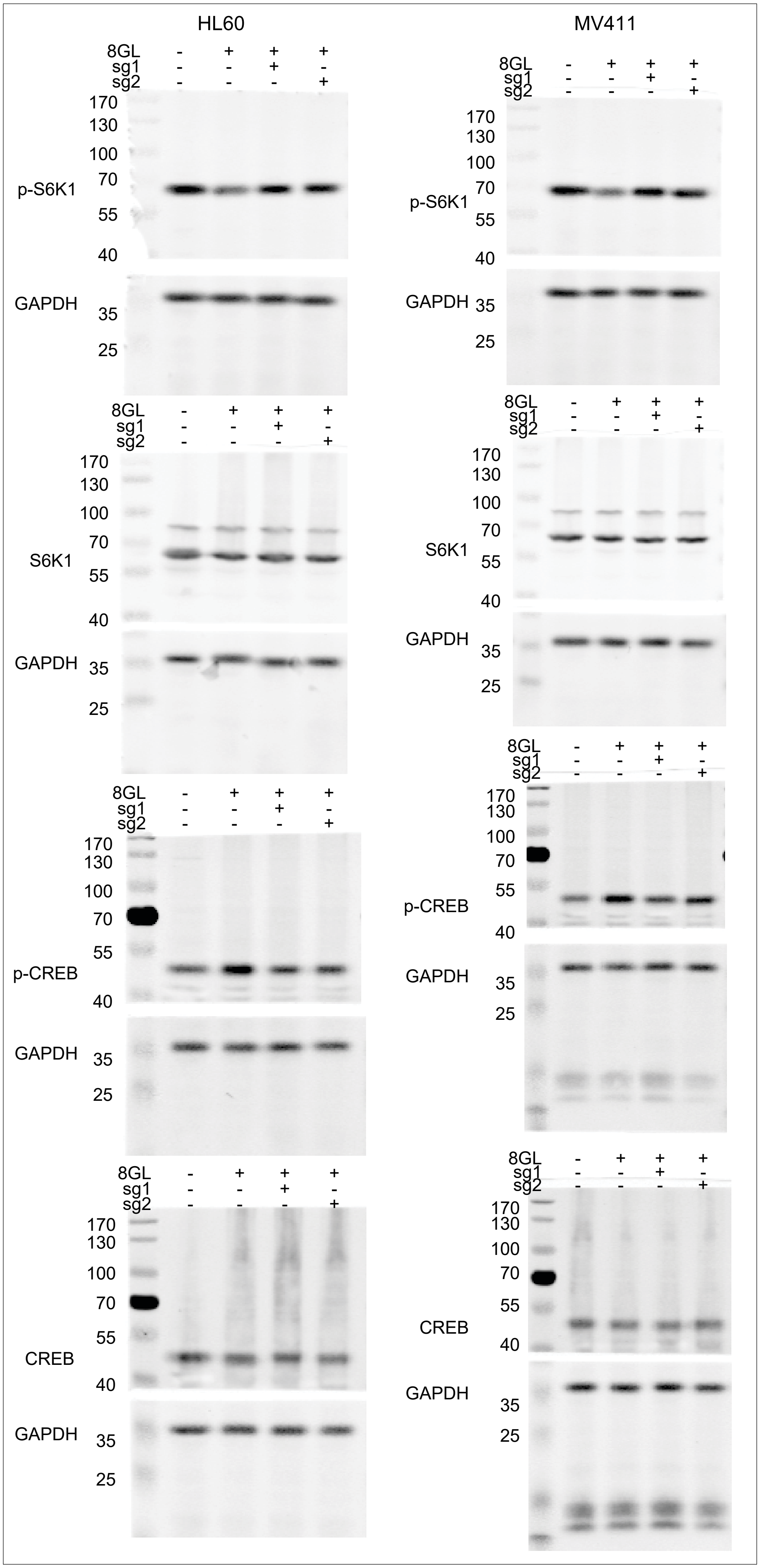

HL60
MV411
8GL
sg1
sg2
170
130
100
70
55
40
35
25
p-S6K1
GAPDH
S6K1
p-CREB
CREB

# Figure 5E

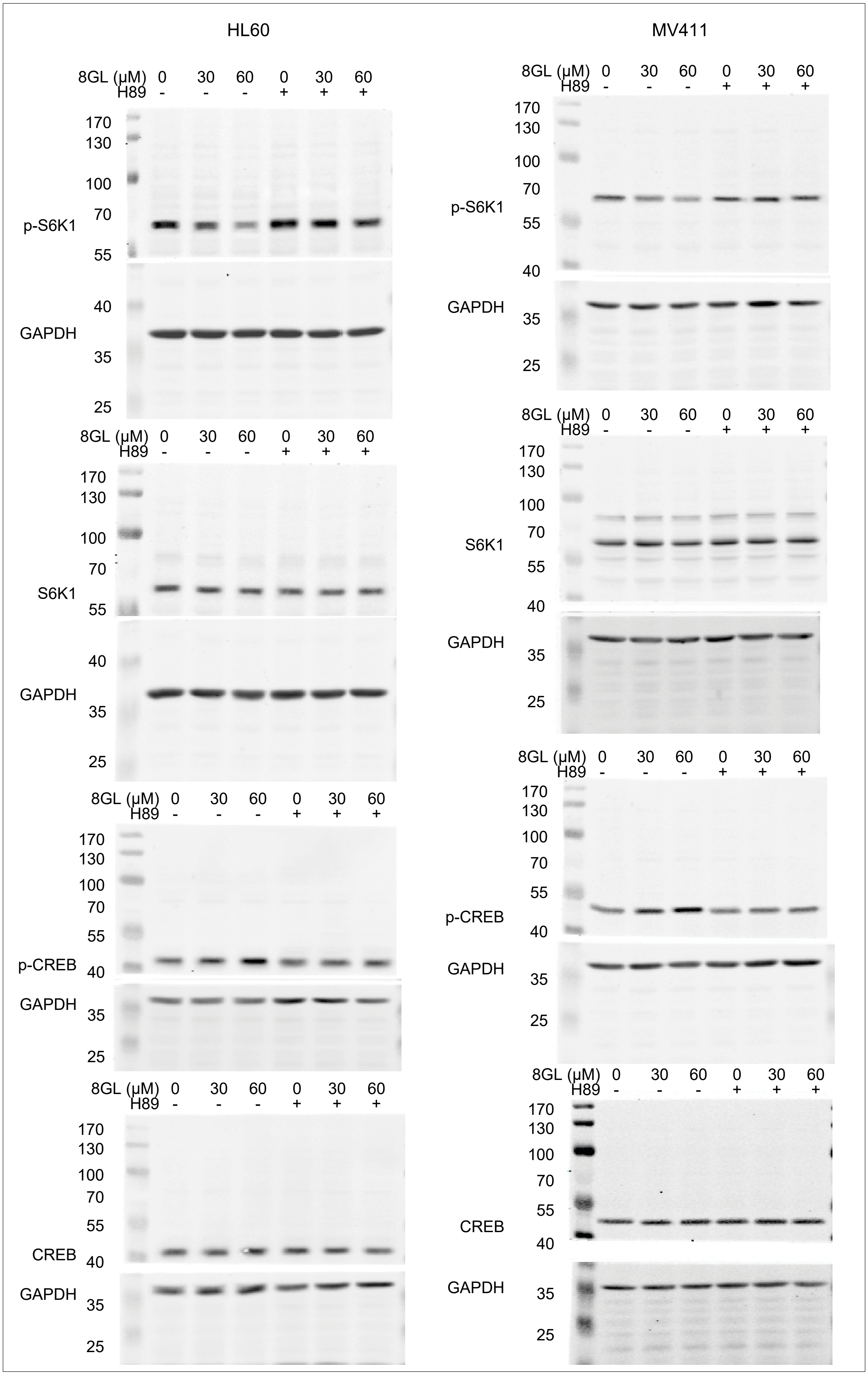
HL60
MV411
8GL (µM) 0 30 60 0 30 60
H89 - - - + + +
170
130
100
70
55
40
35
25
p-S6K1
GAPDH
S6K1
p-CREB
CREB

## Figure 5F

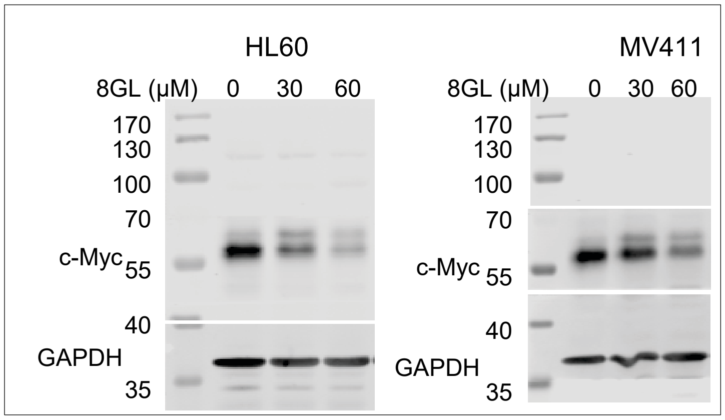


## Figure 5G

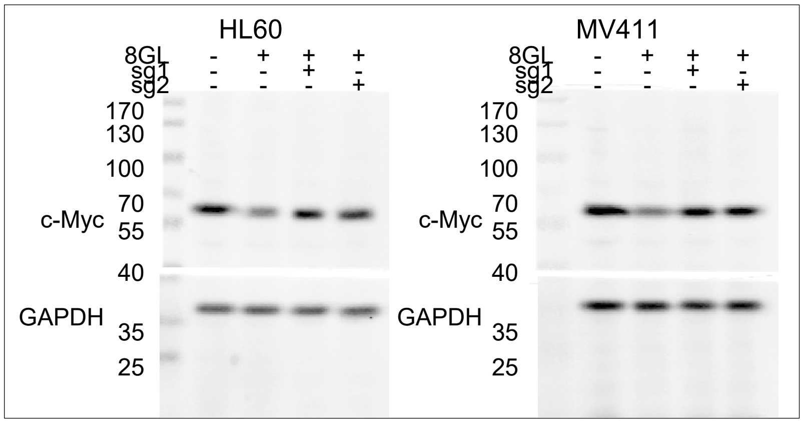

# Figure S2D

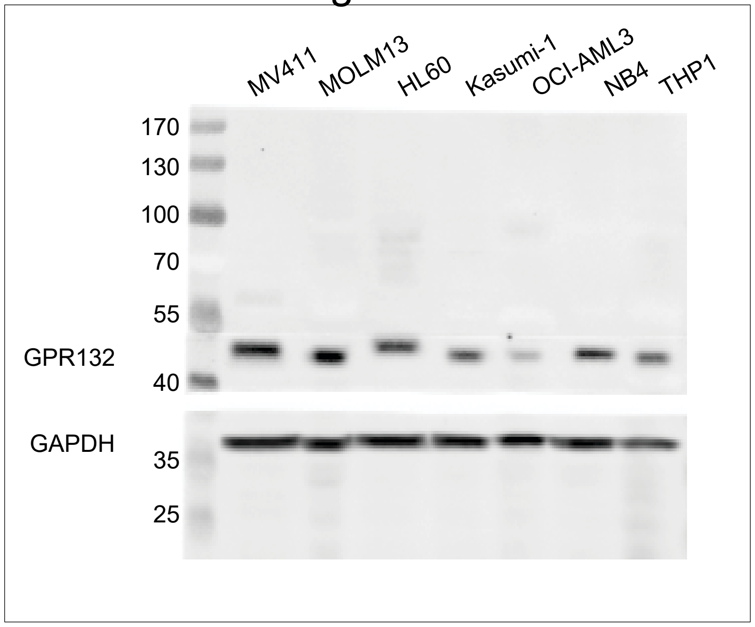


# Figure S2F

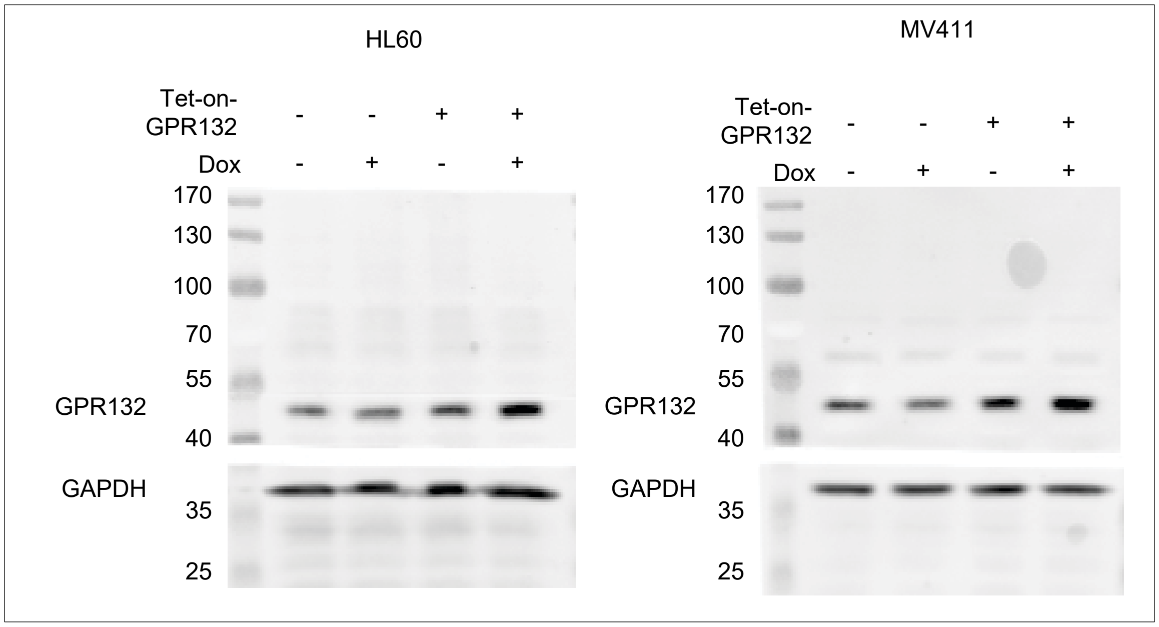

Figure S5A

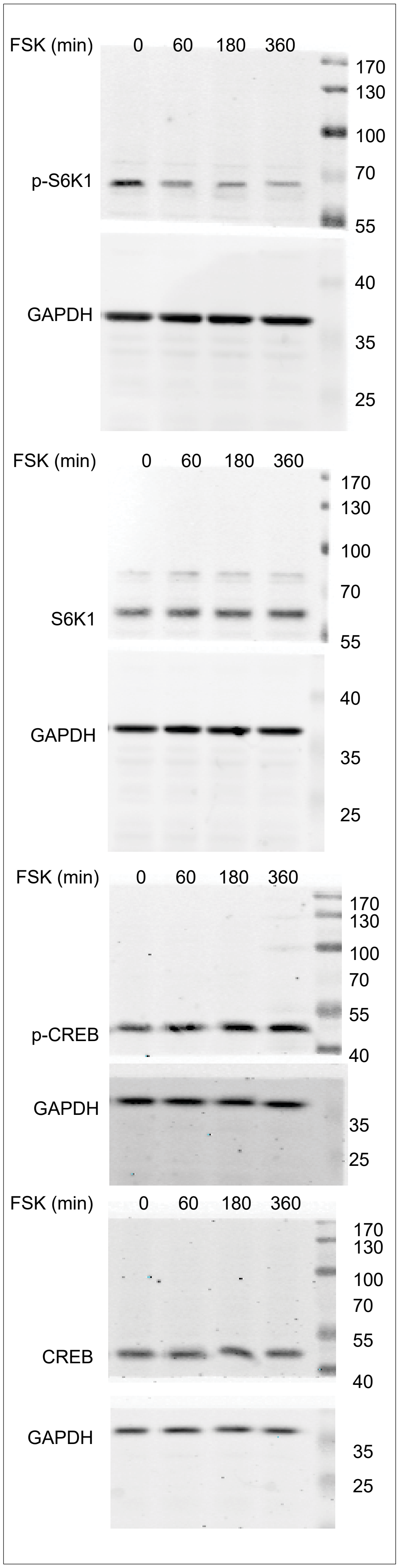


Figure S5B

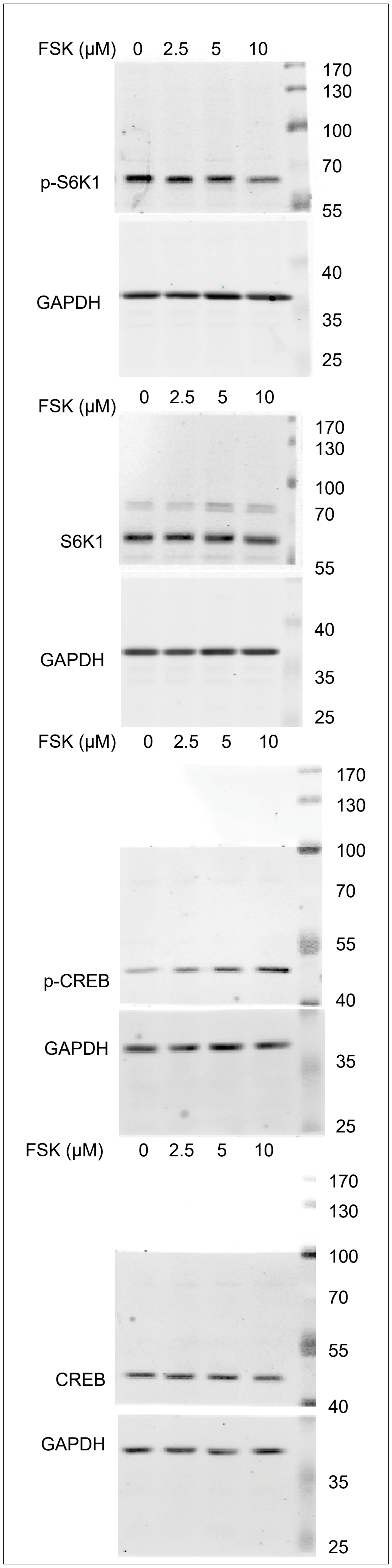

# Figure S5C

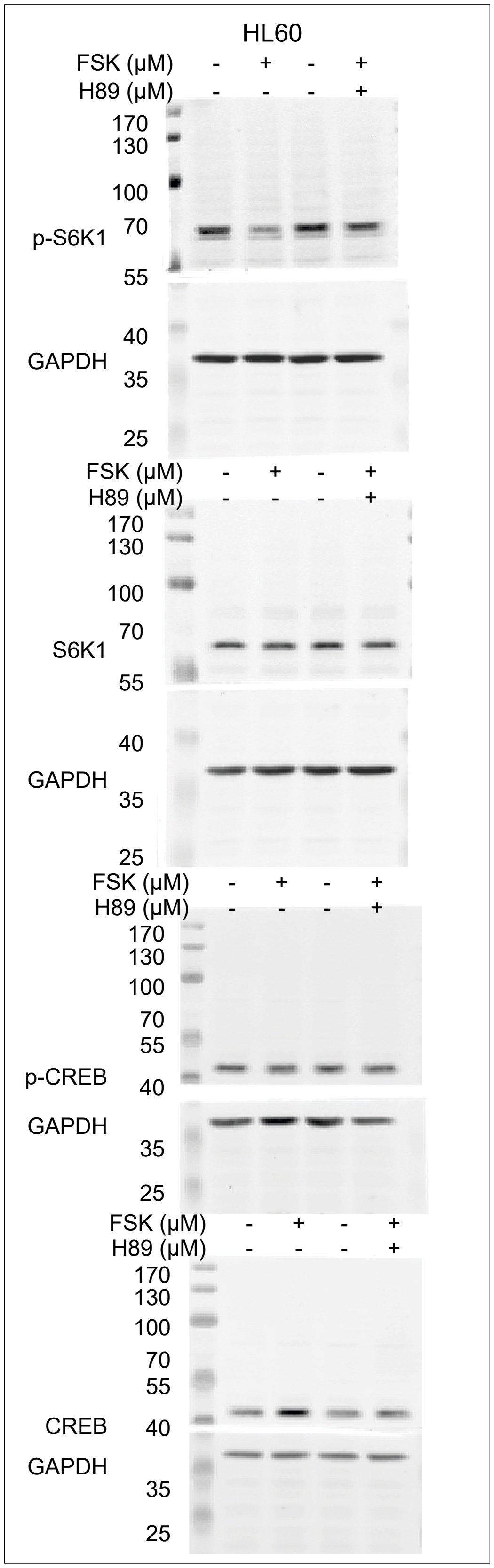


# Figure S5D

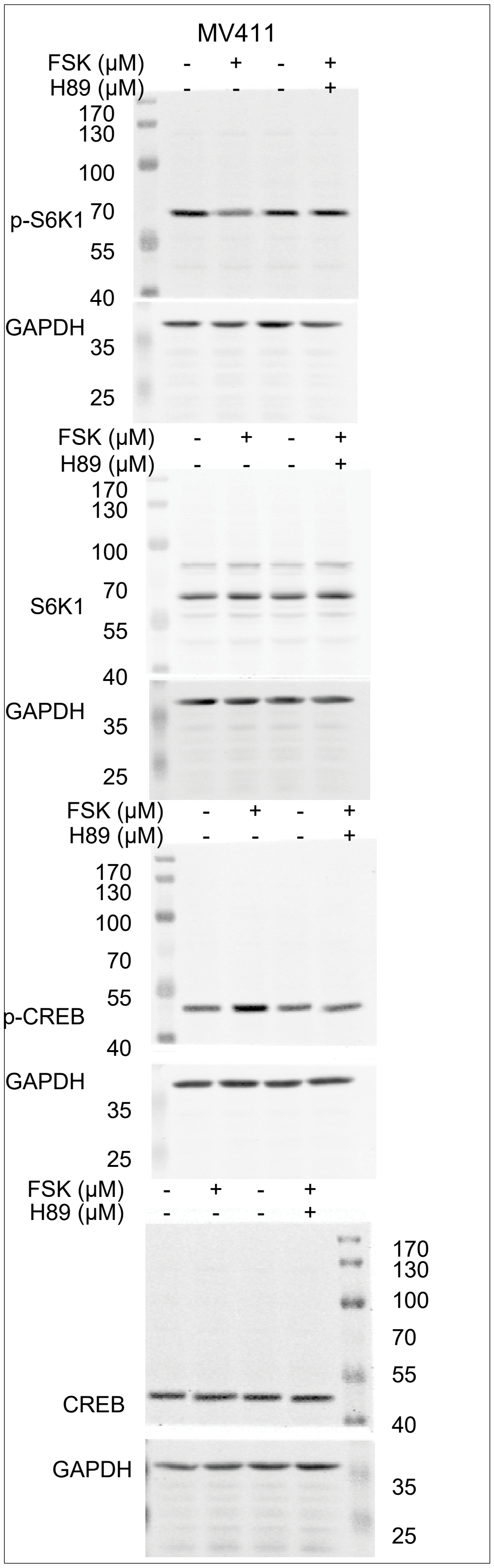

# Figure S6E

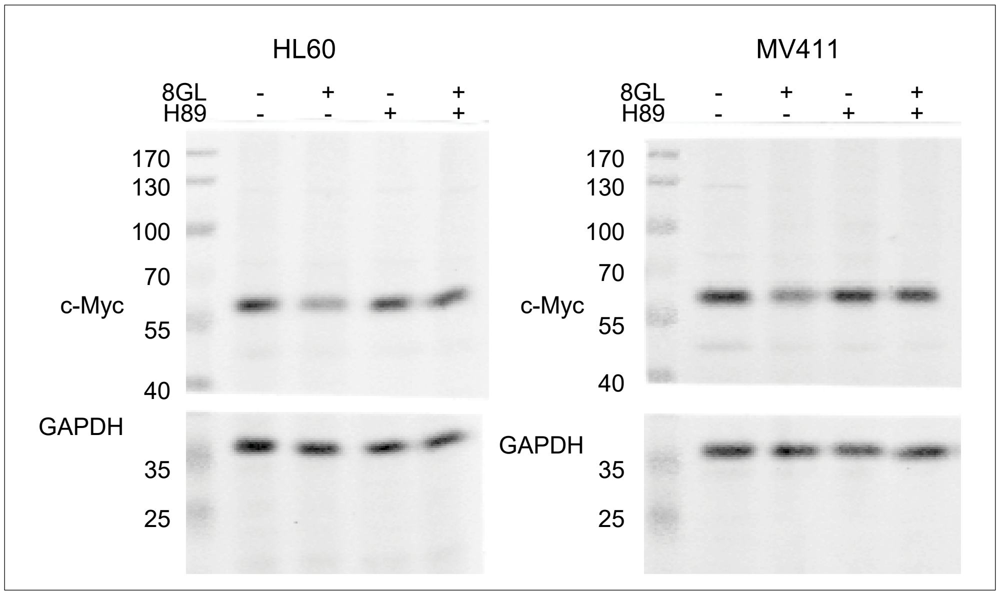

# Figure S7E

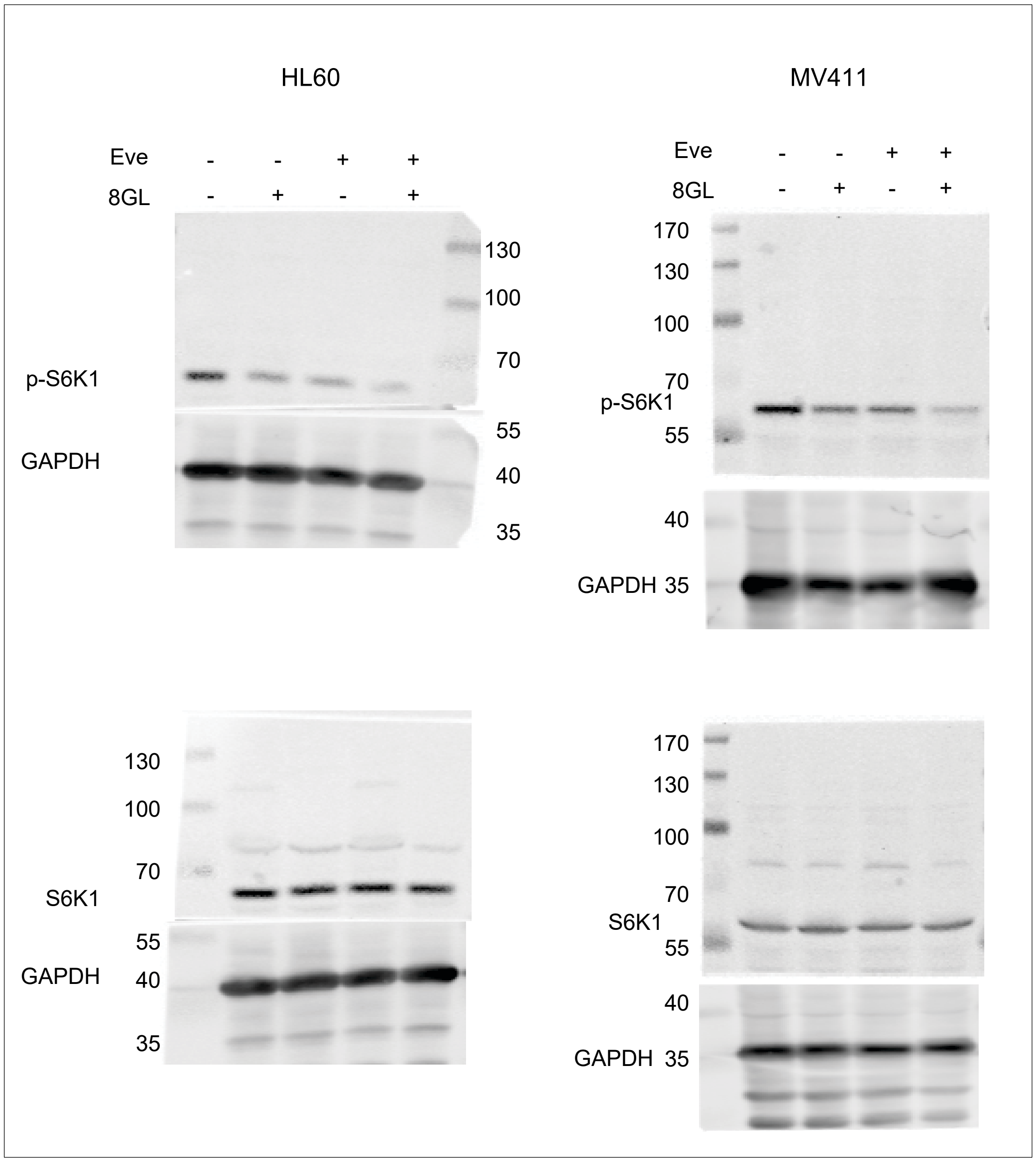

Supplement: Supplementary file 2 — Full length western blots [file 41419_2022_5434_MOESM2_ESM.pdf]
